# Supplementary figures and images for: Enhanced local feature extraction of lite network with scale-invariant CNN for precise segmentation of small brain tumors in MRI (part 2 of 4)
Source: PLoS One. 2025 Oct 28;20(10):e0334447. doi: 10.1371/journal.pone.0334447 (PMC12561956; doi:10.1371/journal.pone.0334447)

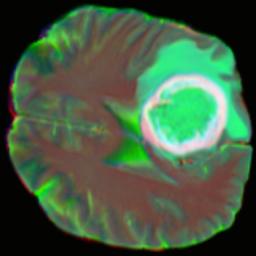

Supplement: S1 Dataset — (ZIP) [file pone.0334447.s001.zip › train/image/00156.jpg]

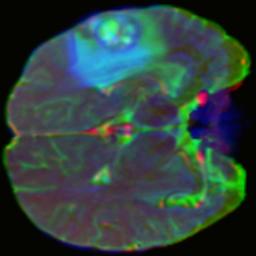

Supplement: S1 Dataset — (ZIP) [file pone.0334447.s001.zip › train/image/00157.jpg]

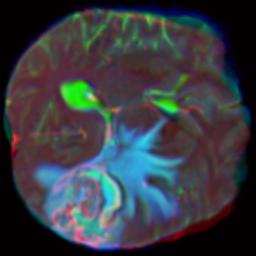

Supplement: S1 Dataset — (ZIP) [file pone.0334447.s001.zip › train/image/00158.jpg]

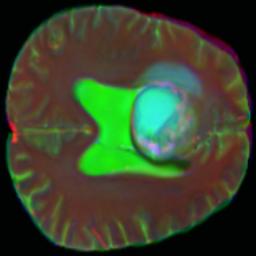

Supplement: S1 Dataset — (ZIP) [file pone.0334447.s001.zip › train/image/00159.jpg]

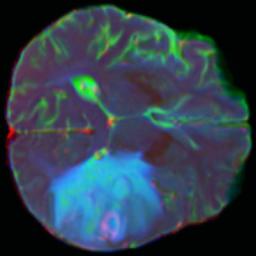

Supplement: S1 Dataset — (ZIP) [file pone.0334447.s001.zip › train/image/00160.jpg]

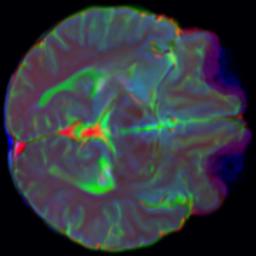

Supplement: S1 Dataset — (ZIP) [file pone.0334447.s001.zip › train/image/00162.jpg]

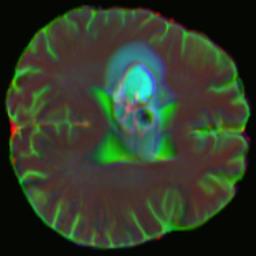

Supplement: S1 Dataset — (ZIP) [file pone.0334447.s001.zip › train/image/00165.jpg]

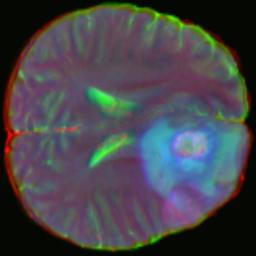

Supplement: S1 Dataset — (ZIP) [file pone.0334447.s001.zip › train/image/00166.jpg]

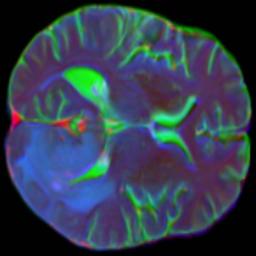

Supplement: S1 Dataset — (ZIP) [file pone.0334447.s001.zip › train/image/00167.jpg]

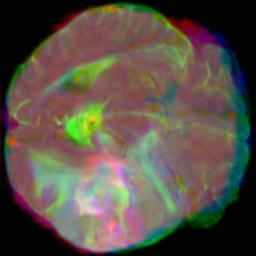

Supplement: S1 Dataset — (ZIP) [file pone.0334447.s001.zip › train/image/00169.jpg]

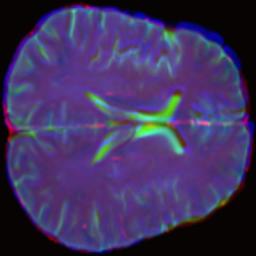

Supplement: S1 Dataset — (ZIP) [file pone.0334447.s001.zip › train/image/00170.jpg]

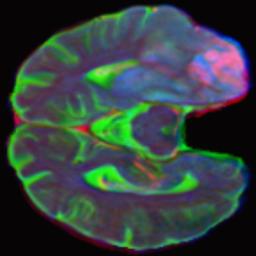

Supplement: S1 Dataset — (ZIP) [file pone.0334447.s001.zip › train/image/00171.jpg]

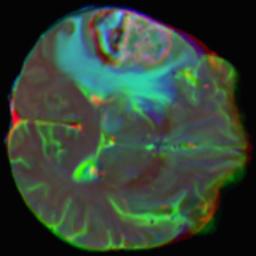

Supplement: S1 Dataset — (ZIP) [file pone.0334447.s001.zip › train/image/00172.jpg]

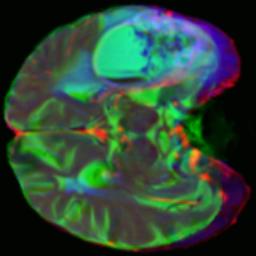

Supplement: S1 Dataset — (ZIP) [file pone.0334447.s001.zip › train/image/00176.jpg]

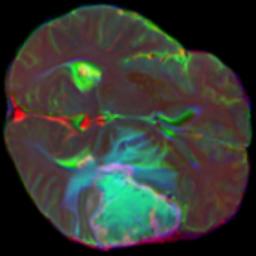

Supplement: S1 Dataset — (ZIP) [file pone.0334447.s001.zip › train/image/00177.jpg]

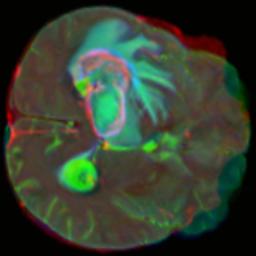

Supplement: S1 Dataset — (ZIP) [file pone.0334447.s001.zip › train/image/00178.jpg]

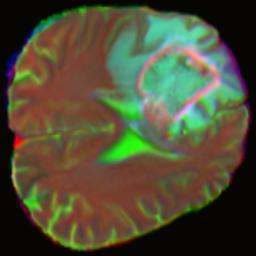

Supplement: S1 Dataset — (ZIP) [file pone.0334447.s001.zip › train/image/00183.jpg]

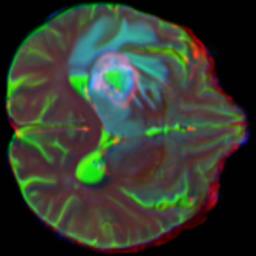

Supplement: S1 Dataset — (ZIP) [file pone.0334447.s001.zip › train/image/00184.jpg]

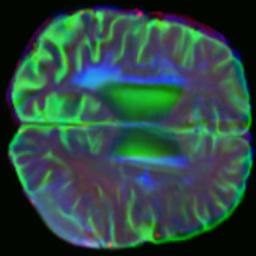

Supplement: S1 Dataset — (ZIP) [file pone.0334447.s001.zip › train/image/00185.jpg]

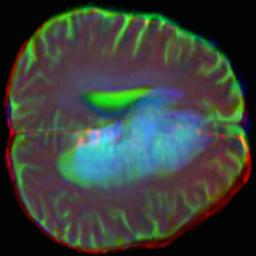

Supplement: S1 Dataset — (ZIP) [file pone.0334447.s001.zip › train/image/00186.jpg]

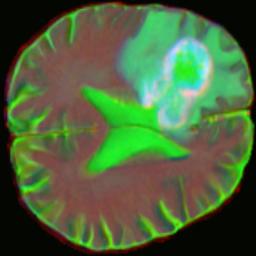

Supplement: S1 Dataset — (ZIP) [file pone.0334447.s001.zip › train/image/00187.jpg]

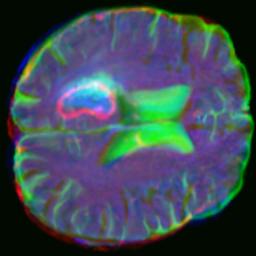

Supplement: S1 Dataset — (ZIP) [file pone.0334447.s001.zip › train/image/00188.jpg]

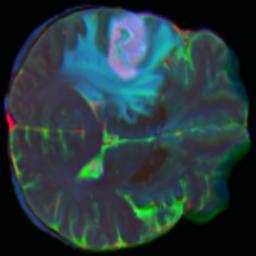

Supplement: S1 Dataset — (ZIP) [file pone.0334447.s001.zip › train/image/00191.jpg]

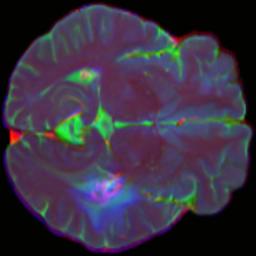

Supplement: S1 Dataset — (ZIP) [file pone.0334447.s001.zip › train/image/00192.jpg]

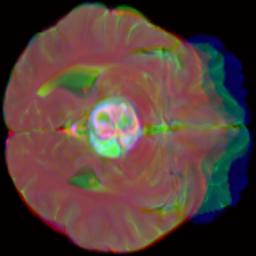

Supplement: S1 Dataset — (ZIP) [file pone.0334447.s001.zip › train/image/00193.jpg]

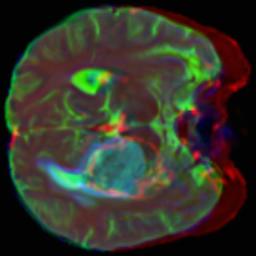

Supplement: S1 Dataset — (ZIP) [file pone.0334447.s001.zip › train/image/00194.jpg]

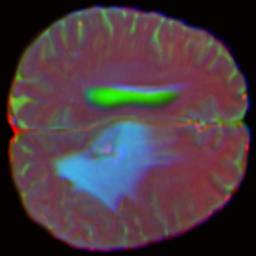

Supplement: S1 Dataset — (ZIP) [file pone.0334447.s001.zip › train/image/00195.jpg]

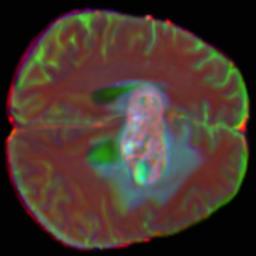

Supplement: S1 Dataset — (ZIP) [file pone.0334447.s001.zip › train/image/00196.jpg]

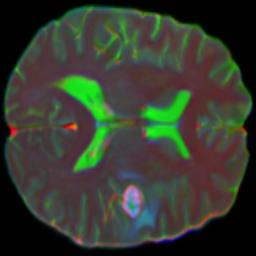

Supplement: S1 Dataset — (ZIP) [file pone.0334447.s001.zip › train/image/00197.jpg]

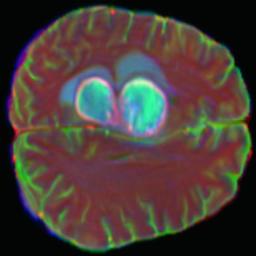

Supplement: S1 Dataset — (ZIP) [file pone.0334447.s001.zip › train/image/00199.jpg]

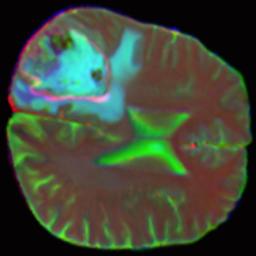

Supplement: S1 Dataset — (ZIP) [file pone.0334447.s001.zip › train/image/00201.jpg]

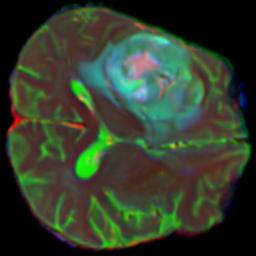

Supplement: S1 Dataset — (ZIP) [file pone.0334447.s001.zip › train/image/00203.jpg]

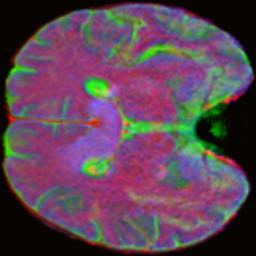

Supplement: S1 Dataset — (ZIP) [file pone.0334447.s001.zip › train/image/00204.jpg]

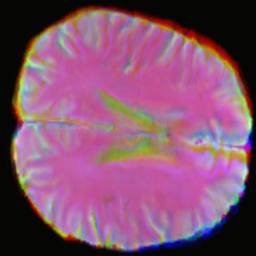

Supplement: S1 Dataset — (ZIP) [file pone.0334447.s001.zip › train/image/00206.jpg]

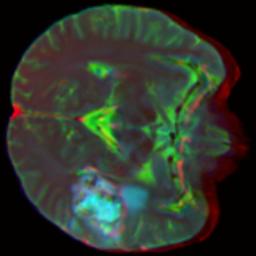

Supplement: S1 Dataset — (ZIP) [file pone.0334447.s001.zip › train/image/00209.jpg]

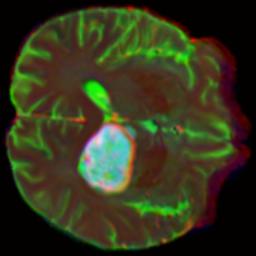

Supplement: S1 Dataset — (ZIP) [file pone.0334447.s001.zip › train/image/00210.jpg]

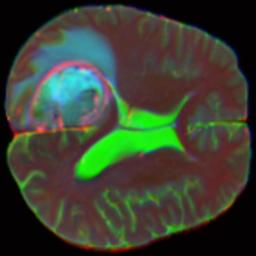

Supplement: S1 Dataset — (ZIP) [file pone.0334447.s001.zip › train/image/00211.jpg]

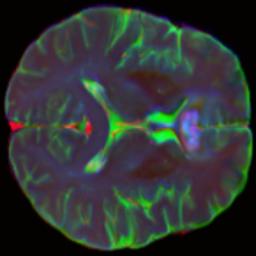

Supplement: S1 Dataset — (ZIP) [file pone.0334447.s001.zip › train/image/00212.jpg]

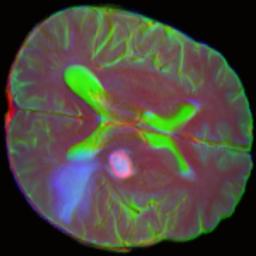

Supplement: S1 Dataset — (ZIP) [file pone.0334447.s001.zip › train/image/00214.jpg]

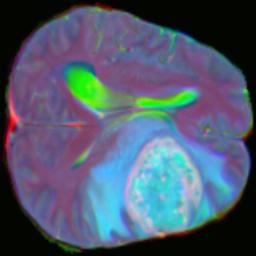

Supplement: S1 Dataset — (ZIP) [file pone.0334447.s001.zip › train/image/00216.jpg]

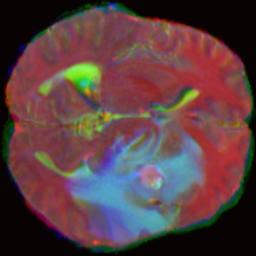

Supplement: S1 Dataset — (ZIP) [file pone.0334447.s001.zip › train/image/00217.jpg]

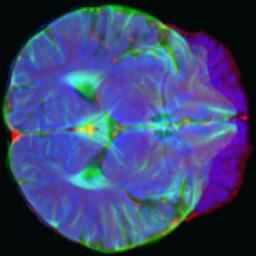

Supplement: S1 Dataset — (ZIP) [file pone.0334447.s001.zip › train/image/00218.jpg]

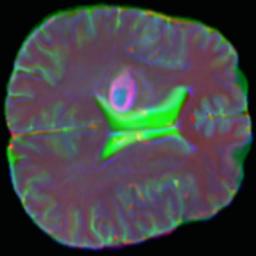

Supplement: S1 Dataset — (ZIP) [file pone.0334447.s001.zip › train/image/00219.jpg]

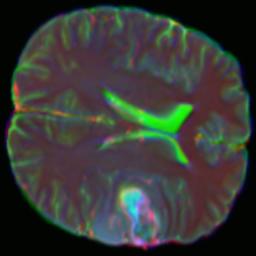

Supplement: S1 Dataset — (ZIP) [file pone.0334447.s001.zip › train/image/00220.jpg]

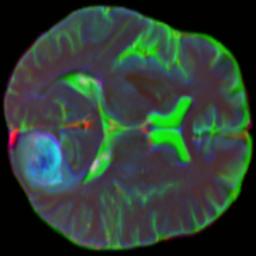

Supplement: S1 Dataset — (ZIP) [file pone.0334447.s001.zip › train/image/00221.jpg]

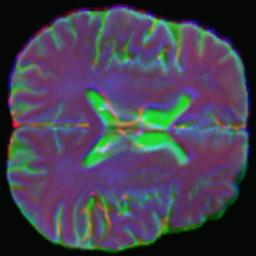

Supplement: S1 Dataset — (ZIP) [file pone.0334447.s001.zip › train/image/00222.jpg]

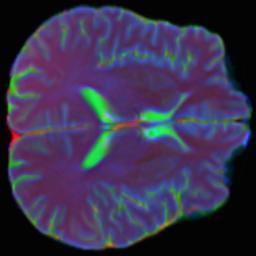

Supplement: S1 Dataset — (ZIP) [file pone.0334447.s001.zip › train/image/00227.jpg]

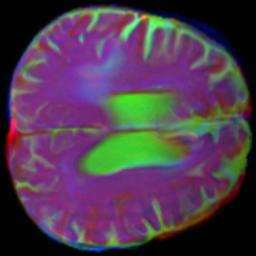

Supplement: S1 Dataset — (ZIP) [file pone.0334447.s001.zip › train/image/00228.jpg]

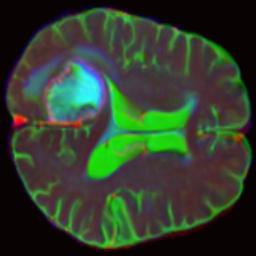

Supplement: S1 Dataset — (ZIP) [file pone.0334447.s001.zip › train/image/00230.jpg]

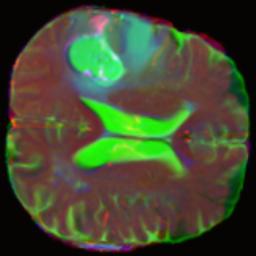

Supplement: S1 Dataset — (ZIP) [file pone.0334447.s001.zip › train/image/00231.jpg]

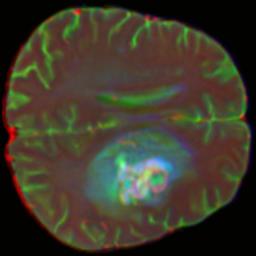

Supplement: S1 Dataset — (ZIP) [file pone.0334447.s001.zip › train/image/00233.jpg]

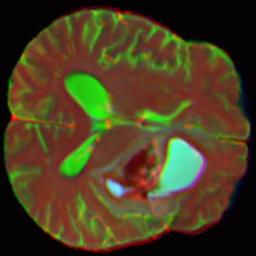

Supplement: S1 Dataset — (ZIP) [file pone.0334447.s001.zip › train/image/00234.jpg]

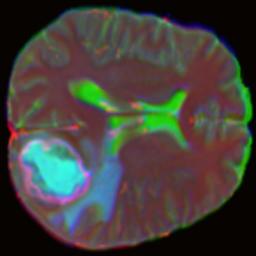

Supplement: S1 Dataset — (ZIP) [file pone.0334447.s001.zip › train/image/00235.jpg]

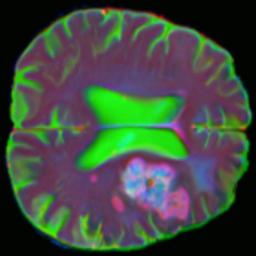

Supplement: S1 Dataset — (ZIP) [file pone.0334447.s001.zip › train/image/00236.jpg]

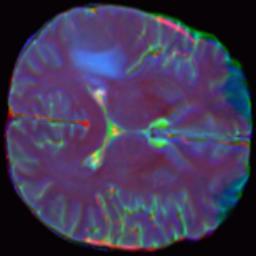

Supplement: S1 Dataset — (ZIP) [file pone.0334447.s001.zip › train/image/00237.jpg]

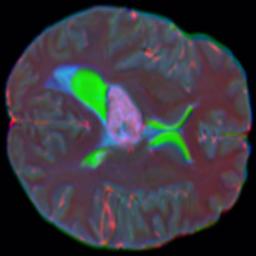

Supplement: S1 Dataset — (ZIP) [file pone.0334447.s001.zip › train/image/00238.jpg]

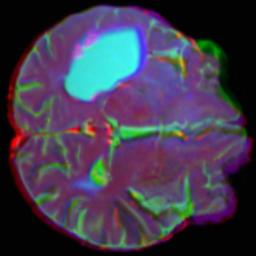

Supplement: S1 Dataset — (ZIP) [file pone.0334447.s001.zip › train/image/00239.jpg]

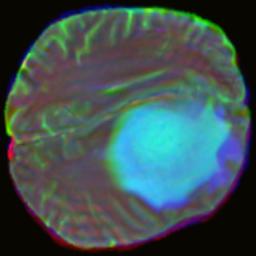

Supplement: S1 Dataset — (ZIP) [file pone.0334447.s001.zip › train/image/00240.jpg]

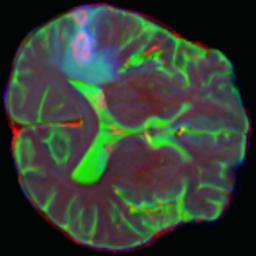

Supplement: S1 Dataset — (ZIP) [file pone.0334447.s001.zip › train/image/00241.jpg]

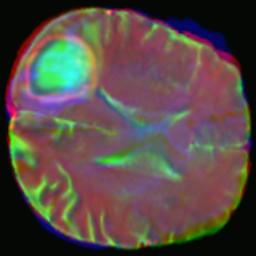

Supplement: S1 Dataset — (ZIP) [file pone.0334447.s001.zip › train/image/00242.jpg]

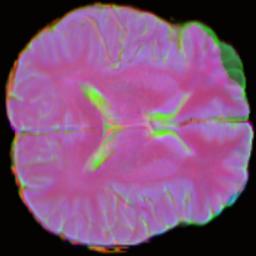

Supplement: S1 Dataset — (ZIP) [file pone.0334447.s001.zip › train/image/00243.jpg]

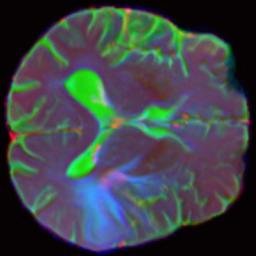

Supplement: S1 Dataset — (ZIP) [file pone.0334447.s001.zip › train/image/00245.jpg]

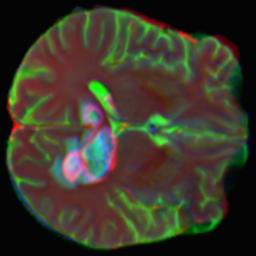

Supplement: S1 Dataset — (ZIP) [file pone.0334447.s001.zip › train/image/00246.jpg]

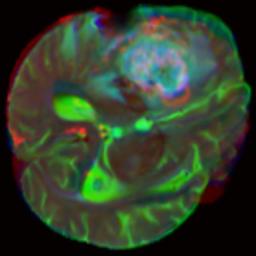

Supplement: S1 Dataset — (ZIP) [file pone.0334447.s001.zip › train/image/00247.jpg]

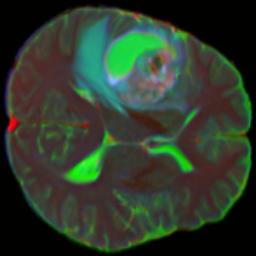

Supplement: S1 Dataset — (ZIP) [file pone.0334447.s001.zip › train/image/00249.jpg]

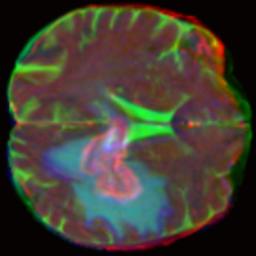

Supplement: S1 Dataset — (ZIP) [file pone.0334447.s001.zip › train/image/00250.jpg]

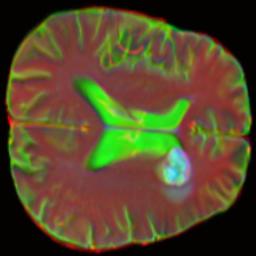

Supplement: S1 Dataset — (ZIP) [file pone.0334447.s001.zip › train/image/00251.jpg]

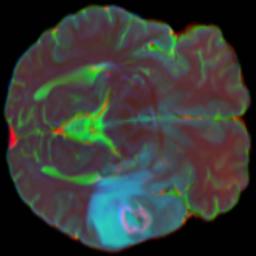

Supplement: S1 Dataset — (ZIP) [file pone.0334447.s001.zip › train/image/00253.jpg]

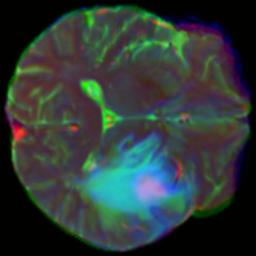

Supplement: S1 Dataset — (ZIP) [file pone.0334447.s001.zip › train/image/00254.jpg]

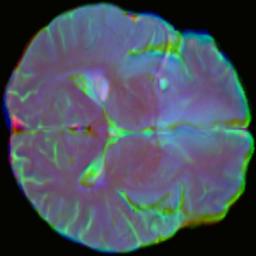

Supplement: S1 Dataset — (ZIP) [file pone.0334447.s001.zip › train/image/00258.jpg]

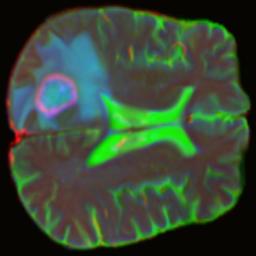

Supplement: S1 Dataset — (ZIP) [file pone.0334447.s001.zip › train/image/00259.jpg]

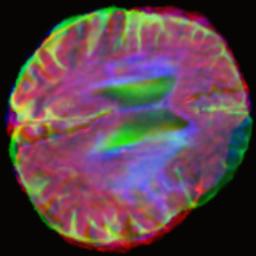

Supplement: S1 Dataset — (ZIP) [file pone.0334447.s001.zip › train/image/00260.jpg]

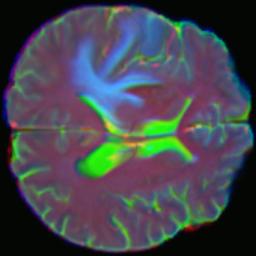

Supplement: S1 Dataset — (ZIP) [file pone.0334447.s001.zip › train/image/00261.jpg]

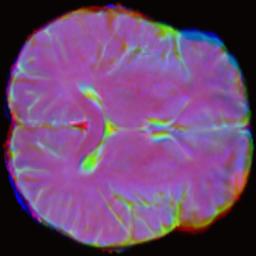

Supplement: S1 Dataset — (ZIP) [file pone.0334447.s001.zip › train/image/00262.jpg]

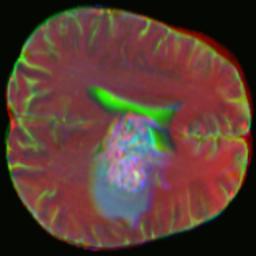

Supplement: S1 Dataset — (ZIP) [file pone.0334447.s001.zip › train/image/00263.jpg]

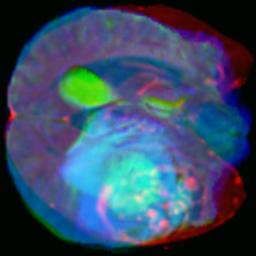

Supplement: S1 Dataset — (ZIP) [file pone.0334447.s001.zip › train/image/00266.jpg]

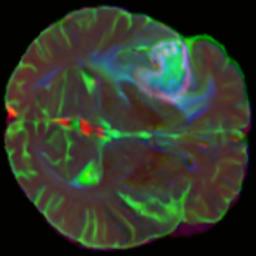

Supplement: S1 Dataset — (ZIP) [file pone.0334447.s001.zip › train/image/00267.jpg]

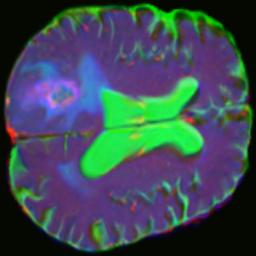

Supplement: S1 Dataset — (ZIP) [file pone.0334447.s001.zip › train/image/00269.jpg]

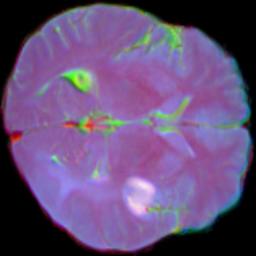

Supplement: S1 Dataset — (ZIP) [file pone.0334447.s001.zip › train/image/00270.jpg]

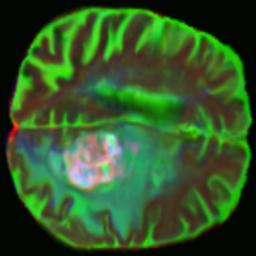

Supplement: S1 Dataset — (ZIP) [file pone.0334447.s001.zip › train/image/00271.jpg]

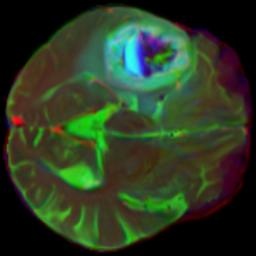

Supplement: S1 Dataset — (ZIP) [file pone.0334447.s001.zip › train/image/00273.jpg]

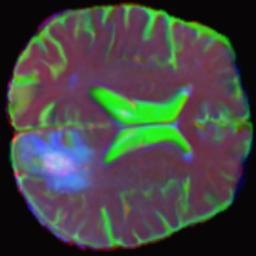

Supplement: S1 Dataset — (ZIP) [file pone.0334447.s001.zip › train/image/00274.jpg]

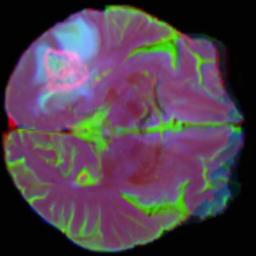

Supplement: S1 Dataset — (ZIP) [file pone.0334447.s001.zip › train/image/00275.jpg]

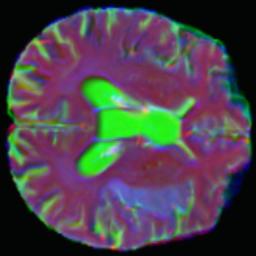

Supplement: S1 Dataset — (ZIP) [file pone.0334447.s001.zip › train/image/00280.jpg]

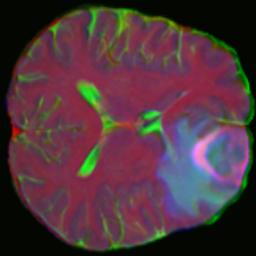

Supplement: S1 Dataset — (ZIP) [file pone.0334447.s001.zip › train/image/00281.jpg]

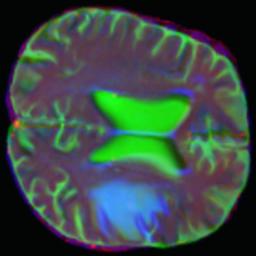

Supplement: S1 Dataset — (ZIP) [file pone.0334447.s001.zip › train/image/00282.jpg]

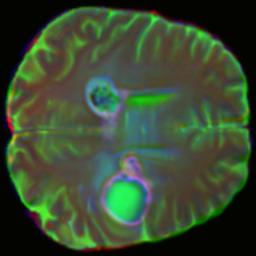

Supplement: S1 Dataset — (ZIP) [file pone.0334447.s001.zip › train/image/00283.jpg]

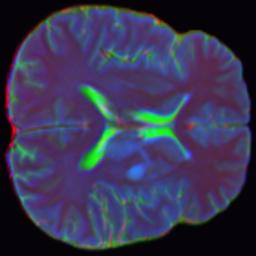

Supplement: S1 Dataset — (ZIP) [file pone.0334447.s001.zip › train/image/00284.jpg]

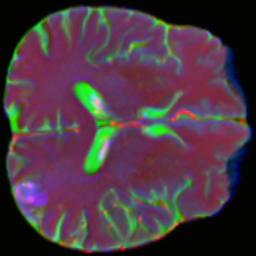

Supplement: S1 Dataset — (ZIP) [file pone.0334447.s001.zip › train/image/00285.jpg]

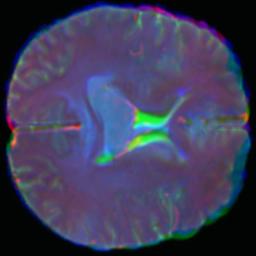

Supplement: S1 Dataset — (ZIP) [file pone.0334447.s001.zip › train/image/00286.jpg]

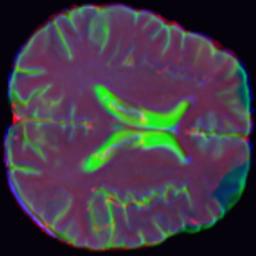

Supplement: S1 Dataset — (ZIP) [file pone.0334447.s001.zip › train/image/00288.jpg]

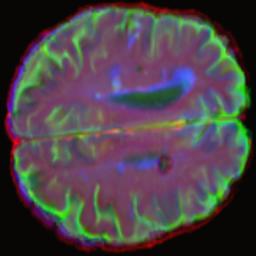

Supplement: S1 Dataset — (ZIP) [file pone.0334447.s001.zip › train/image/00289.jpg]

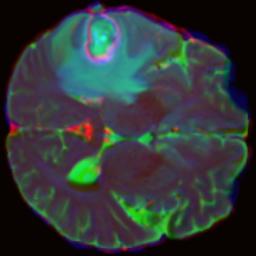

Supplement: S1 Dataset — (ZIP) [file pone.0334447.s001.zip › train/image/00290.jpg]

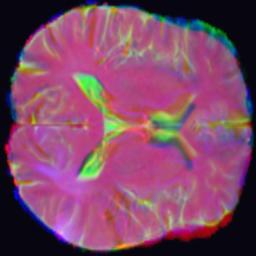

Supplement: S1 Dataset — (ZIP) [file pone.0334447.s001.zip › train/image/00291.jpg]

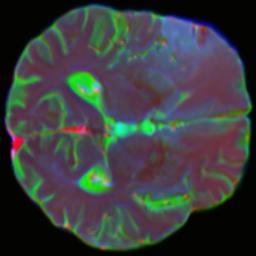

Supplement: S1 Dataset — (ZIP) [file pone.0334447.s001.zip › train/image/00293.jpg]

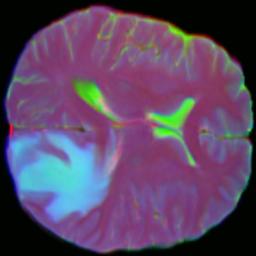

Supplement: S1 Dataset — (ZIP) [file pone.0334447.s001.zip › train/image/00294.jpg]

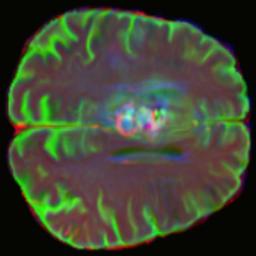

Supplement: S1 Dataset — (ZIP) [file pone.0334447.s001.zip › train/image/00296.jpg]

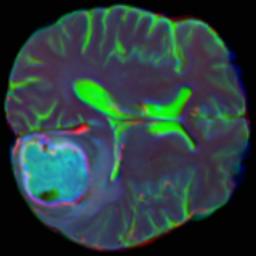

Supplement: S1 Dataset — (ZIP) [file pone.0334447.s001.zip › train/image/00297.jpg]

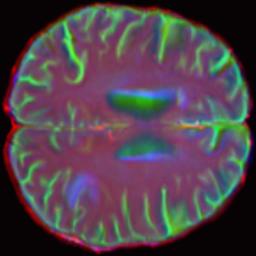

Supplement: S1 Dataset — (ZIP) [file pone.0334447.s001.zip › train/image/00298.jpg]

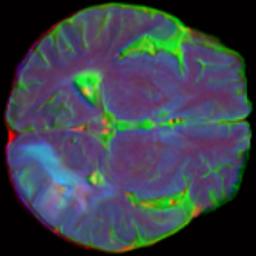

Supplement: S1 Dataset — (ZIP) [file pone.0334447.s001.zip › train/image/00299.jpg]
