# Supplementary figures and images for: Enhanced local feature extraction of lite network with scale-invariant CNN for precise segmentation of small brain tumors in MRI (part 1 of 4)
Source: PLoS One. 2025 Oct 28;20(10):e0334447. doi: 10.1371/journal.pone.0334447 (PMC12561956; doi:10.1371/journal.pone.0334447)

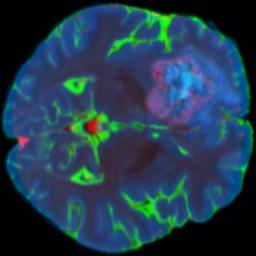

Supplement: S1 Dataset — (ZIP) [file pone.0334447.s001.zip › train/image/00000.jpg]

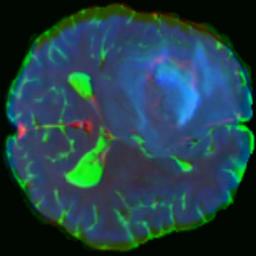

Supplement: S1 Dataset — (ZIP) [file pone.0334447.s001.zip › train/image/00002.jpg]

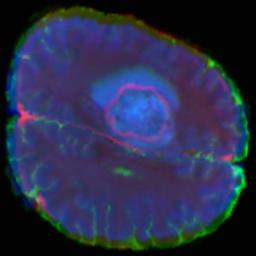

Supplement: S1 Dataset — (ZIP) [file pone.0334447.s001.zip › train/image/00003.jpg]

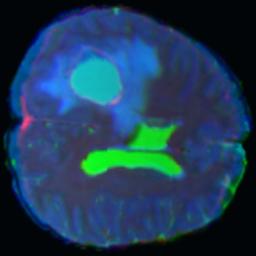

Supplement: S1 Dataset — (ZIP) [file pone.0334447.s001.zip › train/image/00005.jpg]

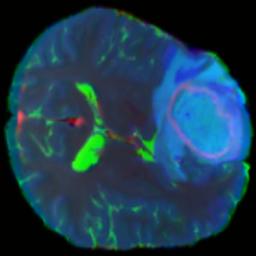

Supplement: S1 Dataset — (ZIP) [file pone.0334447.s001.zip › train/image/00006.jpg]

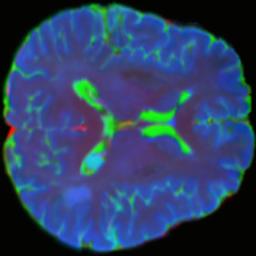

Supplement: S1 Dataset — (ZIP) [file pone.0334447.s001.zip › train/image/00008.jpg]

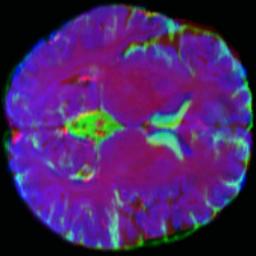

Supplement: S1 Dataset — (ZIP) [file pone.0334447.s001.zip › train/image/00009.jpg]

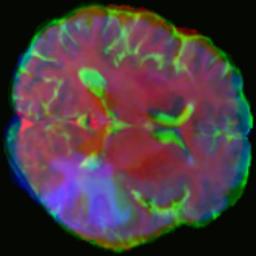

Supplement: S1 Dataset — (ZIP) [file pone.0334447.s001.zip › train/image/00011.jpg]

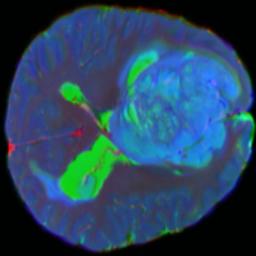

Supplement: S1 Dataset — (ZIP) [file pone.0334447.s001.zip › train/image/00012.jpg]

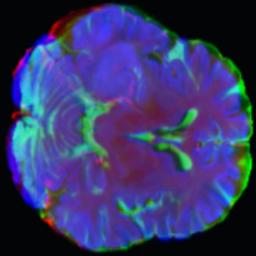

Supplement: S1 Dataset — (ZIP) [file pone.0334447.s001.zip › train/image/00014.jpg]

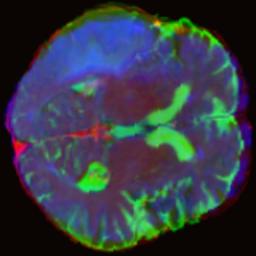

Supplement: S1 Dataset — (ZIP) [file pone.0334447.s001.zip › train/image/00017.jpg]

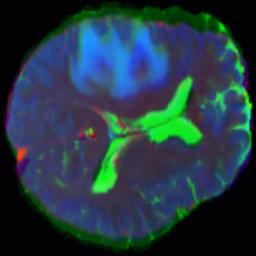

Supplement: S1 Dataset — (ZIP) [file pone.0334447.s001.zip › train/image/00018.jpg]

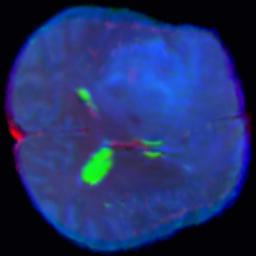

Supplement: S1 Dataset — (ZIP) [file pone.0334447.s001.zip › train/image/00019.jpg]

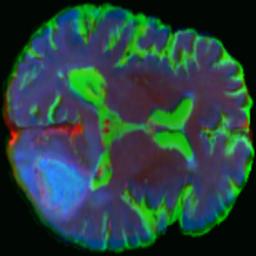

Supplement: S1 Dataset — (ZIP) [file pone.0334447.s001.zip › train/image/00020.jpg]

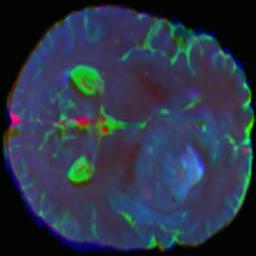

Supplement: S1 Dataset — (ZIP) [file pone.0334447.s001.zip › train/image/00021.jpg]

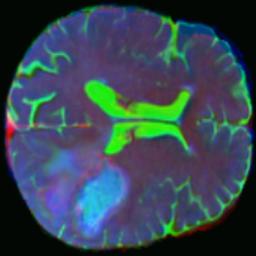

Supplement: S1 Dataset — (ZIP) [file pone.0334447.s001.zip › train/image/00022.jpg]

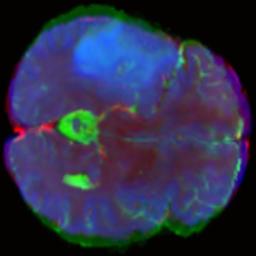

Supplement: S1 Dataset — (ZIP) [file pone.0334447.s001.zip › train/image/00024.jpg]

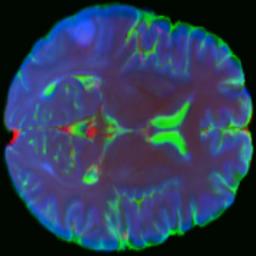

Supplement: S1 Dataset — (ZIP) [file pone.0334447.s001.zip › train/image/00025.jpg]

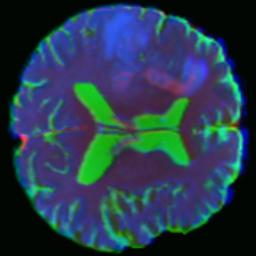

Supplement: S1 Dataset — (ZIP) [file pone.0334447.s001.zip › train/image/00026.jpg]

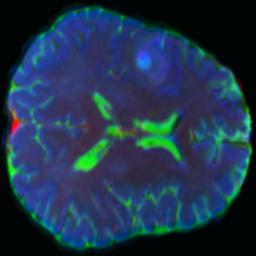

Supplement: S1 Dataset — (ZIP) [file pone.0334447.s001.zip › train/image/00028.jpg]

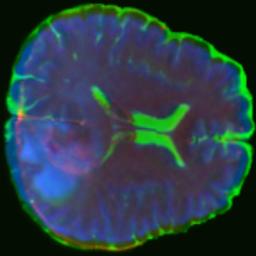

Supplement: S1 Dataset — (ZIP) [file pone.0334447.s001.zip › train/image/00030.jpg]

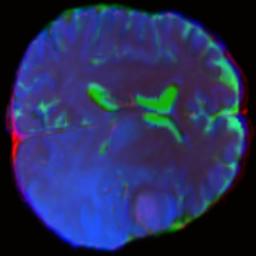

Supplement: S1 Dataset — (ZIP) [file pone.0334447.s001.zip › train/image/00031.jpg]

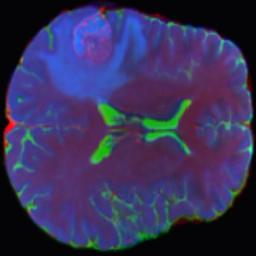

Supplement: S1 Dataset — (ZIP) [file pone.0334447.s001.zip › train/image/00032.jpg]

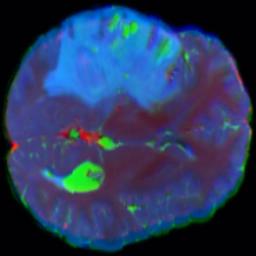

Supplement: S1 Dataset — (ZIP) [file pone.0334447.s001.zip › train/image/00033.jpg]

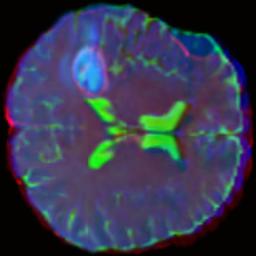

Supplement: S1 Dataset — (ZIP) [file pone.0334447.s001.zip › train/image/00035.jpg]

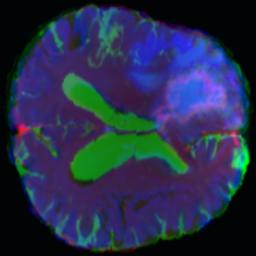

Supplement: S1 Dataset — (ZIP) [file pone.0334447.s001.zip › train/image/00036.jpg]

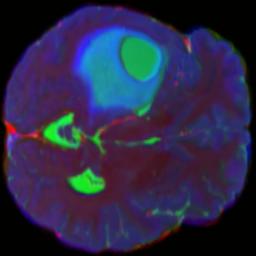

Supplement: S1 Dataset — (ZIP) [file pone.0334447.s001.zip › train/image/00043.jpg]

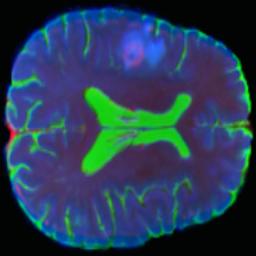

Supplement: S1 Dataset — (ZIP) [file pone.0334447.s001.zip › train/image/00044.jpg]

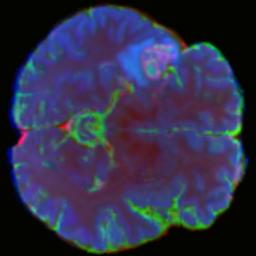

Supplement: S1 Dataset — (ZIP) [file pone.0334447.s001.zip › train/image/00045.jpg]

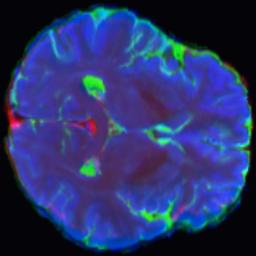

Supplement: S1 Dataset — (ZIP) [file pone.0334447.s001.zip › train/image/00046.jpg]

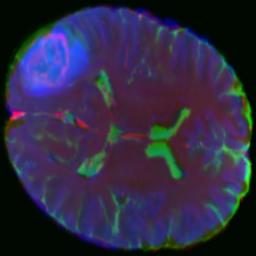

Supplement: S1 Dataset — (ZIP) [file pone.0334447.s001.zip › train/image/00048.jpg]

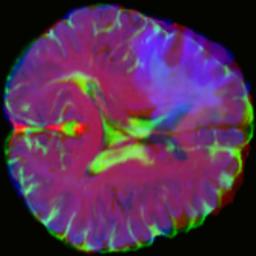

Supplement: S1 Dataset — (ZIP) [file pone.0334447.s001.zip › train/image/00049.jpg]

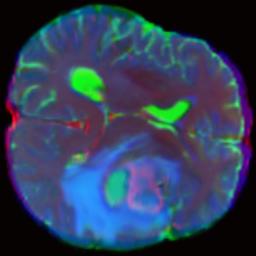

Supplement: S1 Dataset — (ZIP) [file pone.0334447.s001.zip › train/image/00052.jpg]

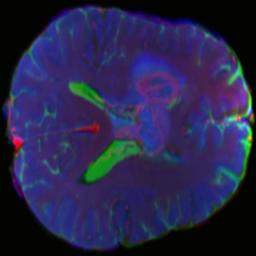

Supplement: S1 Dataset — (ZIP) [file pone.0334447.s001.zip › train/image/00053.jpg]

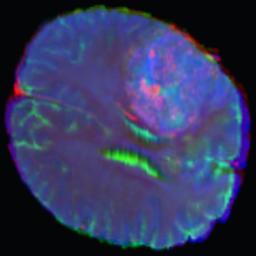

Supplement: S1 Dataset — (ZIP) [file pone.0334447.s001.zip › train/image/00054.jpg]

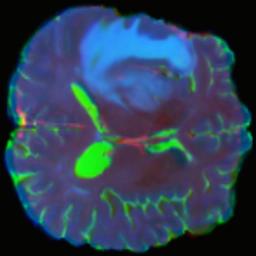

Supplement: S1 Dataset — (ZIP) [file pone.0334447.s001.zip › train/image/00056.jpg]

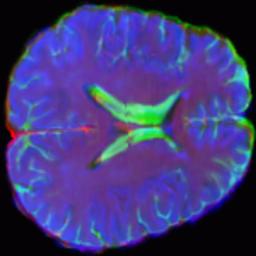

Supplement: S1 Dataset — (ZIP) [file pone.0334447.s001.zip › train/image/00058.jpg]

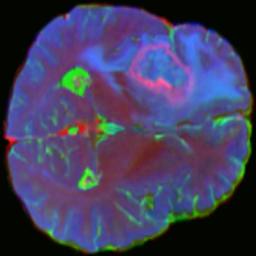

Supplement: S1 Dataset — (ZIP) [file pone.0334447.s001.zip › train/image/00059.jpg]

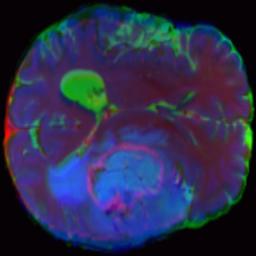

Supplement: S1 Dataset — (ZIP) [file pone.0334447.s001.zip › train/image/00060.jpg]

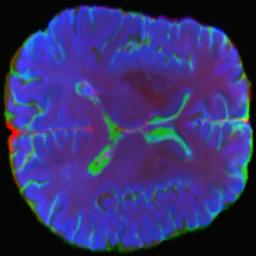

Supplement: S1 Dataset — (ZIP) [file pone.0334447.s001.zip › train/image/00061.jpg]

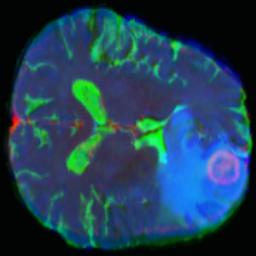

Supplement: S1 Dataset — (ZIP) [file pone.0334447.s001.zip › train/image/00062.jpg]

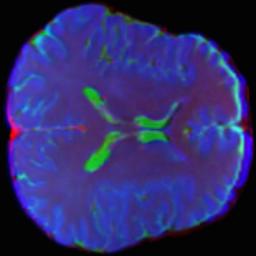

Supplement: S1 Dataset — (ZIP) [file pone.0334447.s001.zip › train/image/00063.jpg]

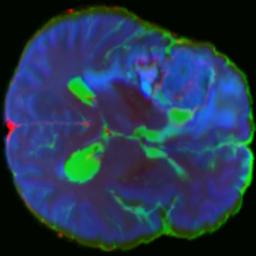

Supplement: S1 Dataset — (ZIP) [file pone.0334447.s001.zip › train/image/00064.jpg]

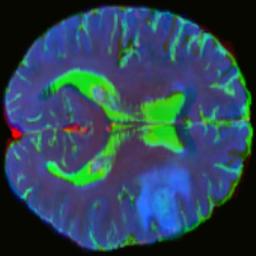

Supplement: S1 Dataset — (ZIP) [file pone.0334447.s001.zip › train/image/00066.jpg]

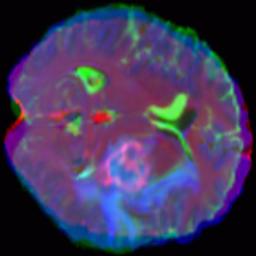

Supplement: S1 Dataset — (ZIP) [file pone.0334447.s001.zip › train/image/00068.jpg]

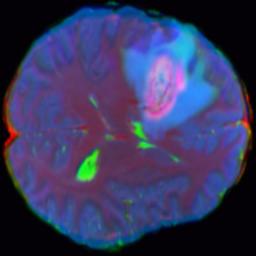

Supplement: S1 Dataset — (ZIP) [file pone.0334447.s001.zip › train/image/00070.jpg]

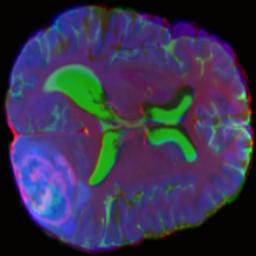

Supplement: S1 Dataset — (ZIP) [file pone.0334447.s001.zip › train/image/00071.jpg]

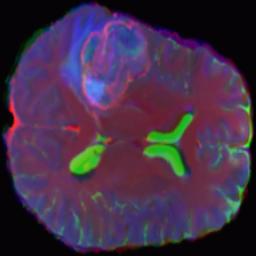

Supplement: S1 Dataset — (ZIP) [file pone.0334447.s001.zip › train/image/00072.jpg]

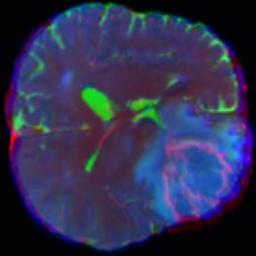

Supplement: S1 Dataset — (ZIP) [file pone.0334447.s001.zip › train/image/00074.jpg]

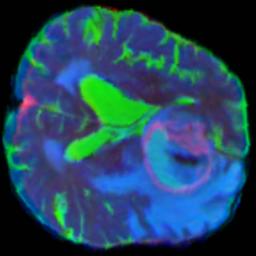

Supplement: S1 Dataset — (ZIP) [file pone.0334447.s001.zip › train/image/00077.jpg]

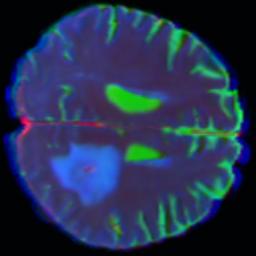

Supplement: S1 Dataset — (ZIP) [file pone.0334447.s001.zip › train/image/00078.jpg]

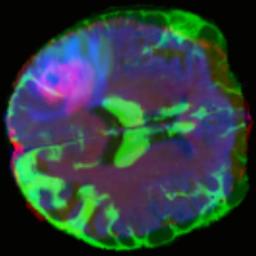

Supplement: S1 Dataset — (ZIP) [file pone.0334447.s001.zip › train/image/00081.jpg]

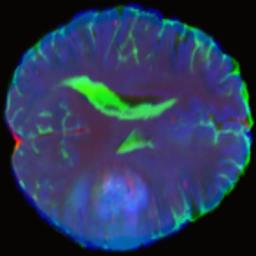

Supplement: S1 Dataset — (ZIP) [file pone.0334447.s001.zip › train/image/00084.jpg]

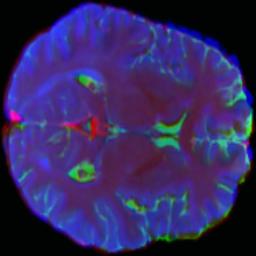

Supplement: S1 Dataset — (ZIP) [file pone.0334447.s001.zip › train/image/00085.jpg]

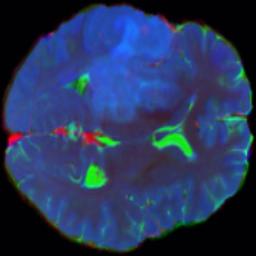

Supplement: S1 Dataset — (ZIP) [file pone.0334447.s001.zip › train/image/00087.jpg]

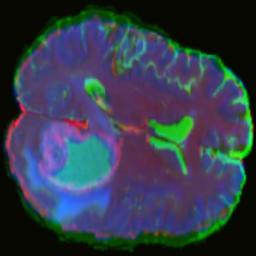

Supplement: S1 Dataset — (ZIP) [file pone.0334447.s001.zip › train/image/00088.jpg]

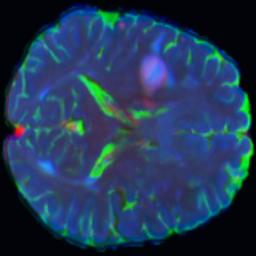

Supplement: S1 Dataset — (ZIP) [file pone.0334447.s001.zip › train/image/00089.jpg]

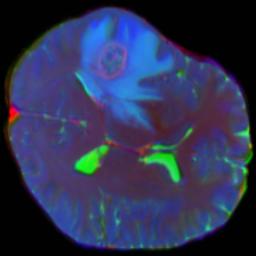

Supplement: S1 Dataset — (ZIP) [file pone.0334447.s001.zip › train/image/00090.jpg]

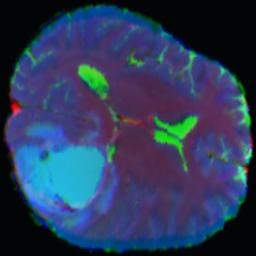

Supplement: S1 Dataset — (ZIP) [file pone.0334447.s001.zip › train/image/00094.jpg]

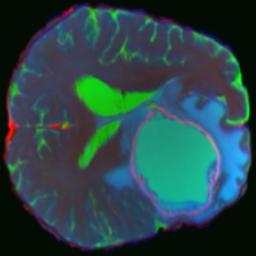

Supplement: S1 Dataset — (ZIP) [file pone.0334447.s001.zip › train/image/00095.jpg]

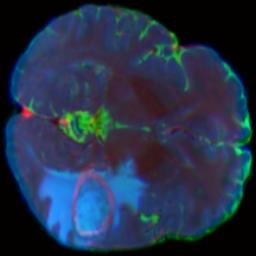

Supplement: S1 Dataset — (ZIP) [file pone.0334447.s001.zip › train/image/00096.jpg]

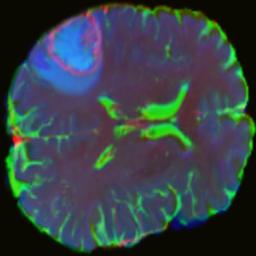

Supplement: S1 Dataset — (ZIP) [file pone.0334447.s001.zip › train/image/00097.jpg]

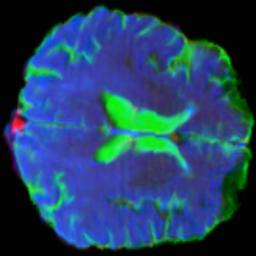

Supplement: S1 Dataset — (ZIP) [file pone.0334447.s001.zip › train/image/00098.jpg]

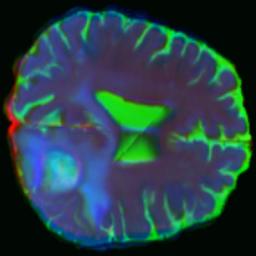

Supplement: S1 Dataset — (ZIP) [file pone.0334447.s001.zip › train/image/00099.jpg]

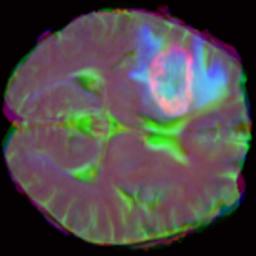

Supplement: S1 Dataset — (ZIP) [file pone.0334447.s001.zip › train/image/00100.jpg]

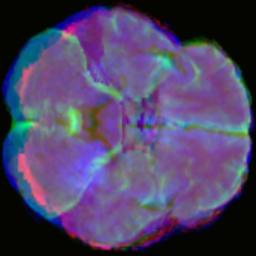

Supplement: S1 Dataset — (ZIP) [file pone.0334447.s001.zip › train/image/00102.jpg]

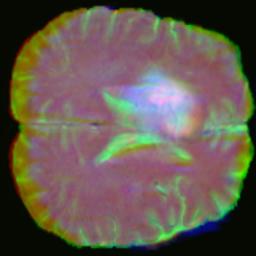

Supplement: S1 Dataset — (ZIP) [file pone.0334447.s001.zip › train/image/00104.jpg]

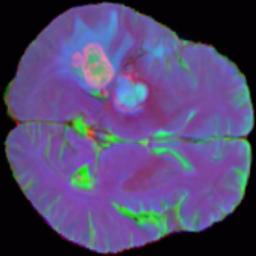

Supplement: S1 Dataset — (ZIP) [file pone.0334447.s001.zip › train/image/00105.jpg]

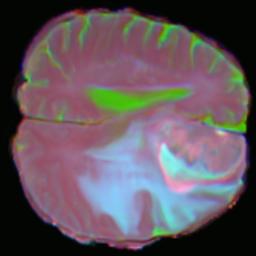

Supplement: S1 Dataset — (ZIP) [file pone.0334447.s001.zip › train/image/00106.jpg]

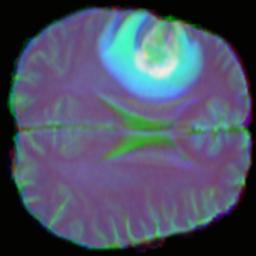

Supplement: S1 Dataset — (ZIP) [file pone.0334447.s001.zip › train/image/00107.jpg]

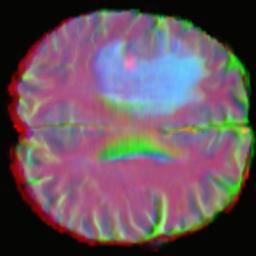

Supplement: S1 Dataset — (ZIP) [file pone.0334447.s001.zip › train/image/00110.jpg]

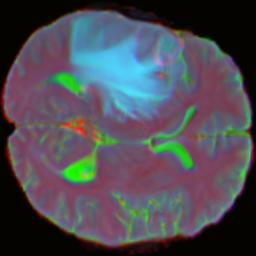

Supplement: S1 Dataset — (ZIP) [file pone.0334447.s001.zip › train/image/00111.jpg]

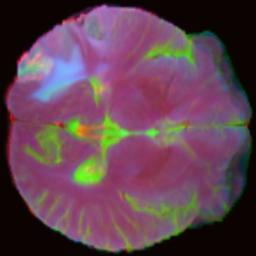

Supplement: S1 Dataset — (ZIP) [file pone.0334447.s001.zip › train/image/00112.jpg]

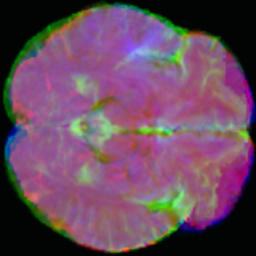

Supplement: S1 Dataset — (ZIP) [file pone.0334447.s001.zip › train/image/00113.jpg]

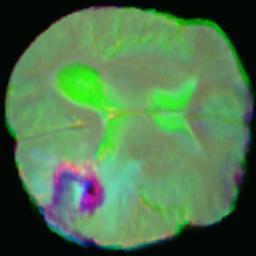

Supplement: S1 Dataset — (ZIP) [file pone.0334447.s001.zip › train/image/00116.jpg]

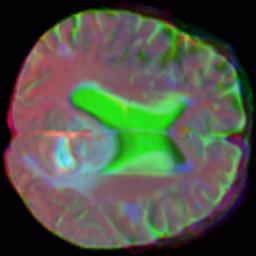

Supplement: S1 Dataset — (ZIP) [file pone.0334447.s001.zip › train/image/00117.jpg]

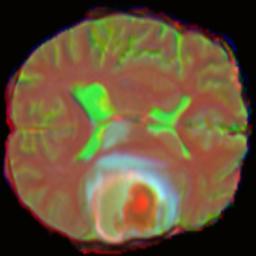

Supplement: S1 Dataset — (ZIP) [file pone.0334447.s001.zip › train/image/00120.jpg]

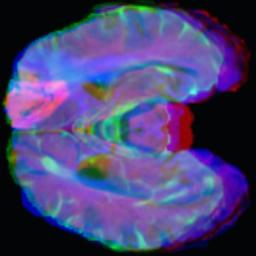

Supplement: S1 Dataset — (ZIP) [file pone.0334447.s001.zip › train/image/00121.jpg]

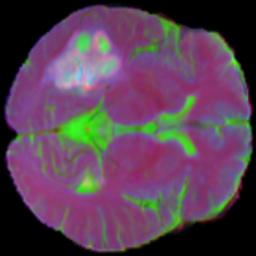

Supplement: S1 Dataset — (ZIP) [file pone.0334447.s001.zip › train/image/00122.jpg]

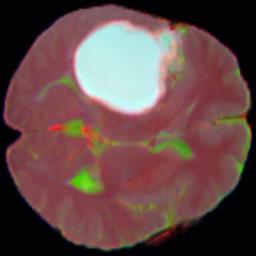

Supplement: S1 Dataset — (ZIP) [file pone.0334447.s001.zip › train/image/00124.jpg]

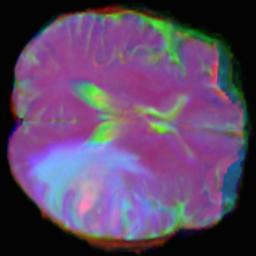

Supplement: S1 Dataset — (ZIP) [file pone.0334447.s001.zip › train/image/00128.jpg]

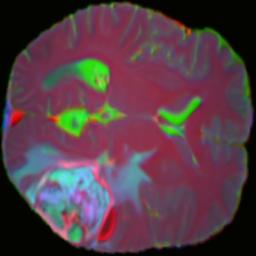

Supplement: S1 Dataset — (ZIP) [file pone.0334447.s001.zip › train/image/00130.jpg]

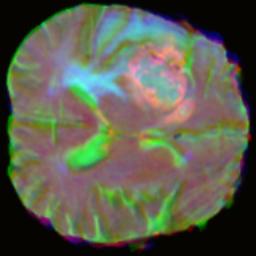

Supplement: S1 Dataset — (ZIP) [file pone.0334447.s001.zip › train/image/00132.jpg]

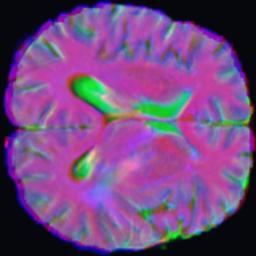

Supplement: S1 Dataset — (ZIP) [file pone.0334447.s001.zip › train/image/00133.jpg]

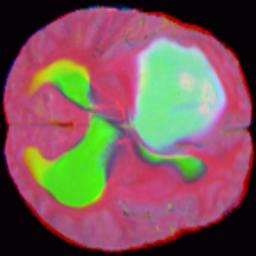

Supplement: S1 Dataset — (ZIP) [file pone.0334447.s001.zip › train/image/00134.jpg]

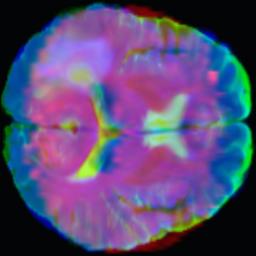

Supplement: S1 Dataset — (ZIP) [file pone.0334447.s001.zip › train/image/00136.jpg]

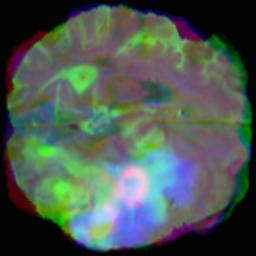

Supplement: S1 Dataset — (ZIP) [file pone.0334447.s001.zip › train/image/00137.jpg]

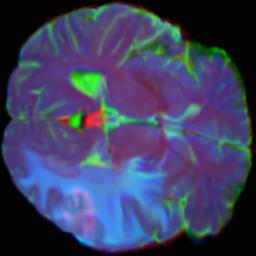

Supplement: S1 Dataset — (ZIP) [file pone.0334447.s001.zip › train/image/00138.jpg]

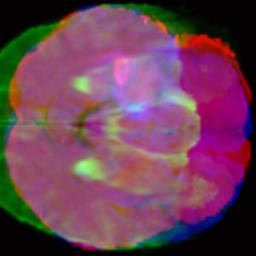

Supplement: S1 Dataset — (ZIP) [file pone.0334447.s001.zip › train/image/00139.jpg]

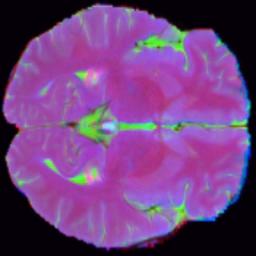

Supplement: S1 Dataset — (ZIP) [file pone.0334447.s001.zip › train/image/00140.jpg]

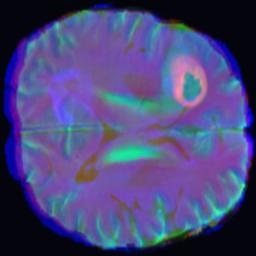

Supplement: S1 Dataset — (ZIP) [file pone.0334447.s001.zip › train/image/00142.jpg]

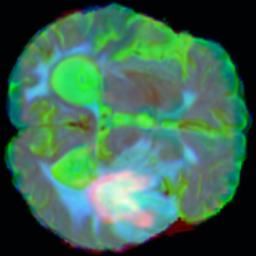

Supplement: S1 Dataset — (ZIP) [file pone.0334447.s001.zip › train/image/00143.jpg]

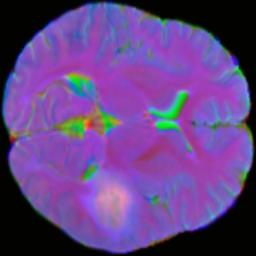

Supplement: S1 Dataset — (ZIP) [file pone.0334447.s001.zip › train/image/00144.jpg]

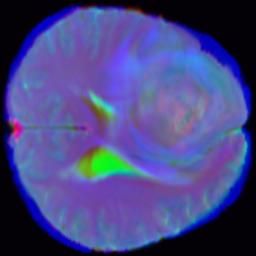

Supplement: S1 Dataset — (ZIP) [file pone.0334447.s001.zip › train/image/00146.jpg]

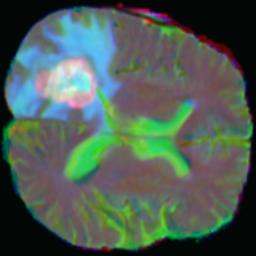

Supplement: S1 Dataset — (ZIP) [file pone.0334447.s001.zip › train/image/00147.jpg]

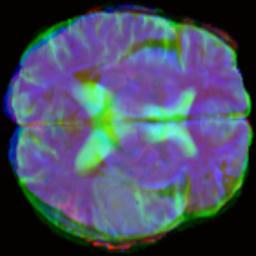

Supplement: S1 Dataset — (ZIP) [file pone.0334447.s001.zip › train/image/00149.jpg]

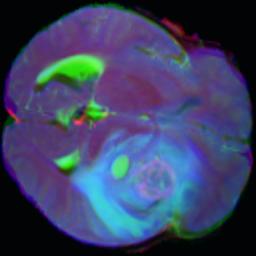

Supplement: S1 Dataset — (ZIP) [file pone.0334447.s001.zip › train/image/00150.jpg]

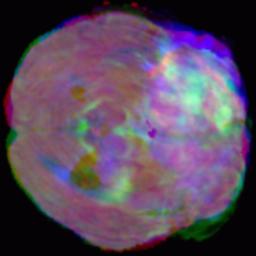

Supplement: S1 Dataset — (ZIP) [file pone.0334447.s001.zip › train/image/00151.jpg]

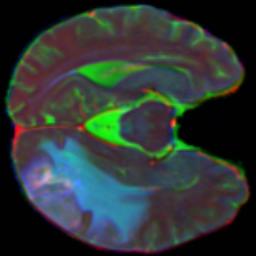

Supplement: S1 Dataset — (ZIP) [file pone.0334447.s001.zip › train/image/00154.jpg]

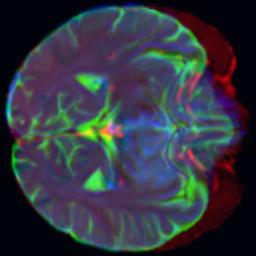

Supplement: S1 Dataset — (ZIP) [file pone.0334447.s001.zip › train/image/00155.jpg]
