# Supplementary figures and images for: Enhanced local feature extraction of lite network with scale-invariant CNN for precise segmentation of small brain tumors in MRI (part 4 of 4)
Source: PLoS One. 2025 Oct 28;20(10):e0334447. doi: 10.1371/journal.pone.0334447 (PMC12561956; doi:10.1371/journal.pone.0334447)

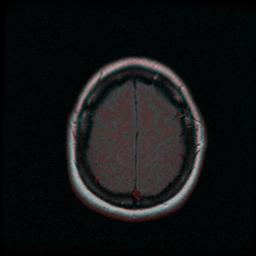

Supplement: S2 Dataset — (ZIP) [file pone.0334447.s002.zip › LGG Segmentation Dataset/train/image/TCGA_DU_6407_19860514_46.jpg]

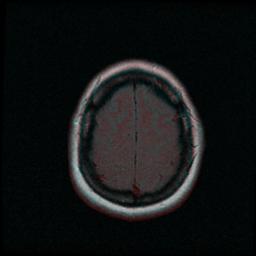

Supplement: S2 Dataset — (ZIP) [file pone.0334447.s002.zip › LGG Segmentation Dataset/train/image/TCGA_DU_6407_19860514_47.jpg]

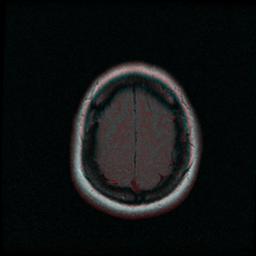

Supplement: S2 Dataset — (ZIP) [file pone.0334447.s002.zip › LGG Segmentation Dataset/train/image/TCGA_DU_6407_19860514_48.jpg]

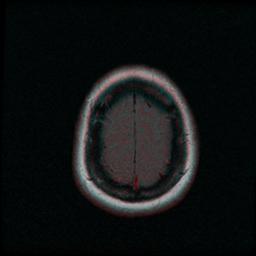

Supplement: S2 Dataset — (ZIP) [file pone.0334447.s002.zip › LGG Segmentation Dataset/train/image/TCGA_DU_6407_19860514_49.jpg]

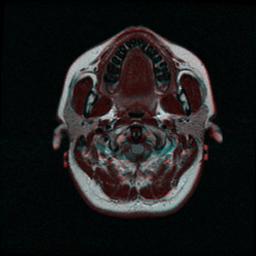

Supplement: S2 Dataset — (ZIP) [file pone.0334447.s002.zip › LGG Segmentation Dataset/train/image/TCGA_DU_6407_19860514_5.jpg]

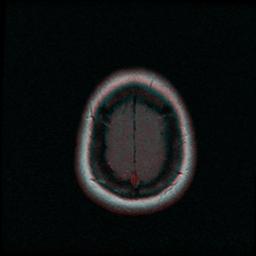

Supplement: S2 Dataset — (ZIP) [file pone.0334447.s002.zip › LGG Segmentation Dataset/train/image/TCGA_DU_6407_19860514_50.jpg]

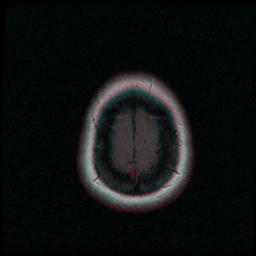

Supplement: S2 Dataset — (ZIP) [file pone.0334447.s002.zip › LGG Segmentation Dataset/train/image/TCGA_DU_6407_19860514_51.jpg]

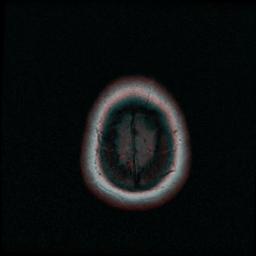

Supplement: S2 Dataset — (ZIP) [file pone.0334447.s002.zip › LGG Segmentation Dataset/train/image/TCGA_DU_6407_19860514_52.jpg]

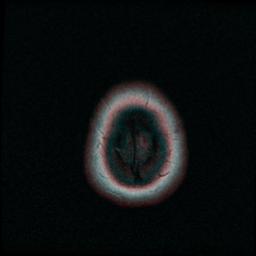

Supplement: S2 Dataset — (ZIP) [file pone.0334447.s002.zip › LGG Segmentation Dataset/train/image/TCGA_DU_6407_19860514_53.jpg]

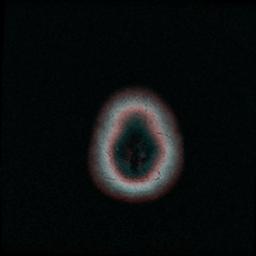

Supplement: S2 Dataset — (ZIP) [file pone.0334447.s002.zip › LGG Segmentation Dataset/train/image/TCGA_DU_6407_19860514_54.jpg]

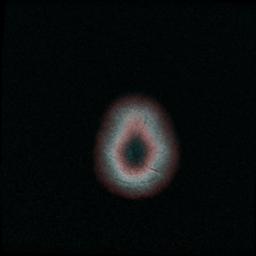

Supplement: S2 Dataset — (ZIP) [file pone.0334447.s002.zip › LGG Segmentation Dataset/train/image/TCGA_DU_6407_19860514_55.jpg]

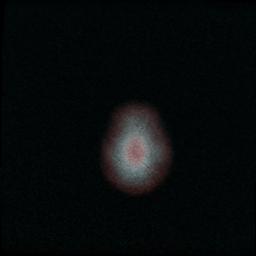

Supplement: S2 Dataset — (ZIP) [file pone.0334447.s002.zip › LGG Segmentation Dataset/train/image/TCGA_DU_6407_19860514_56.jpg]

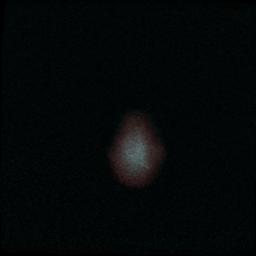

Supplement: S2 Dataset — (ZIP) [file pone.0334447.s002.zip › LGG Segmentation Dataset/train/image/TCGA_DU_6407_19860514_57.jpg]

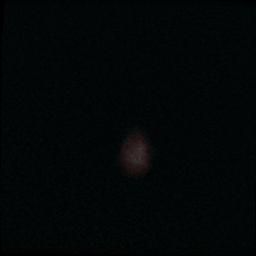

Supplement: S2 Dataset — (ZIP) [file pone.0334447.s002.zip › LGG Segmentation Dataset/train/image/TCGA_DU_6407_19860514_58.jpg]

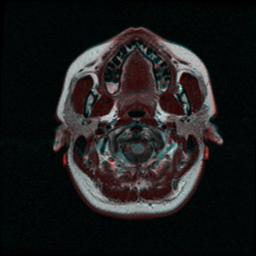

Supplement: S2 Dataset — (ZIP) [file pone.0334447.s002.zip › LGG Segmentation Dataset/train/image/TCGA_DU_6407_19860514_6.jpg]

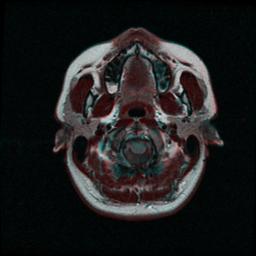

Supplement: S2 Dataset — (ZIP) [file pone.0334447.s002.zip › LGG Segmentation Dataset/train/image/TCGA_DU_6407_19860514_7.jpg]

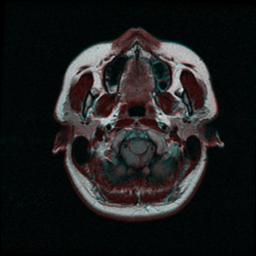

Supplement: S2 Dataset — (ZIP) [file pone.0334447.s002.zip › LGG Segmentation Dataset/train/image/TCGA_DU_6407_19860514_8.jpg]

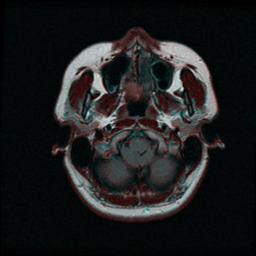

Supplement: S2 Dataset — (ZIP) [file pone.0334447.s002.zip › LGG Segmentation Dataset/train/image/TCGA_DU_6407_19860514_9.jpg]

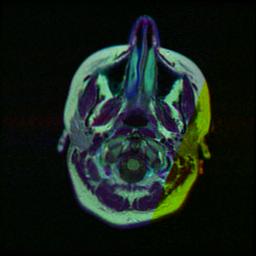

Supplement: S2 Dataset — (ZIP) [file pone.0334447.s002.zip › LGG Segmentation Dataset/train/image/TCGA_DU_6408_19860521_1.jpg]

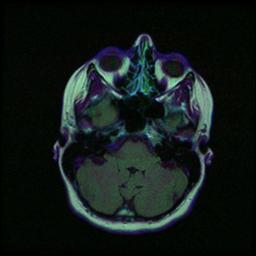

Supplement: S2 Dataset — (ZIP) [file pone.0334447.s002.zip › LGG Segmentation Dataset/train/image/TCGA_DU_6408_19860521_10.jpg]

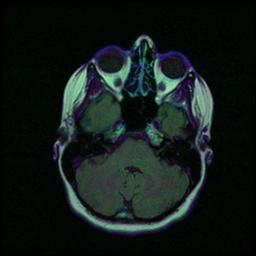

Supplement: S2 Dataset — (ZIP) [file pone.0334447.s002.zip › LGG Segmentation Dataset/train/image/TCGA_DU_6408_19860521_11.jpg]

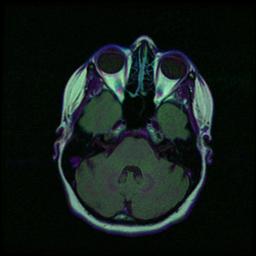

Supplement: S2 Dataset — (ZIP) [file pone.0334447.s002.zip › LGG Segmentation Dataset/train/image/TCGA_DU_6408_19860521_12.jpg]

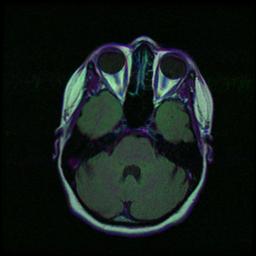

Supplement: S2 Dataset — (ZIP) [file pone.0334447.s002.zip › LGG Segmentation Dataset/train/image/TCGA_DU_6408_19860521_13.jpg]

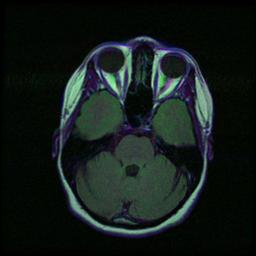

Supplement: S2 Dataset — (ZIP) [file pone.0334447.s002.zip › LGG Segmentation Dataset/train/image/TCGA_DU_6408_19860521_14.jpg]

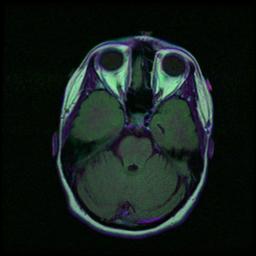

Supplement: S2 Dataset — (ZIP) [file pone.0334447.s002.zip › LGG Segmentation Dataset/train/image/TCGA_DU_6408_19860521_15.jpg]

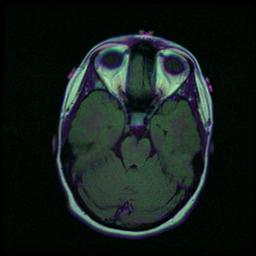

Supplement: S2 Dataset — (ZIP) [file pone.0334447.s002.zip › LGG Segmentation Dataset/train/image/TCGA_DU_6408_19860521_16.jpg]

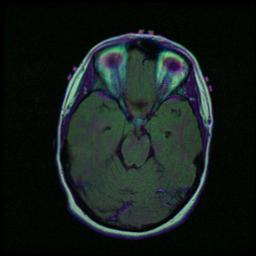

Supplement: S2 Dataset — (ZIP) [file pone.0334447.s002.zip › LGG Segmentation Dataset/train/image/TCGA_DU_6408_19860521_17.jpg]

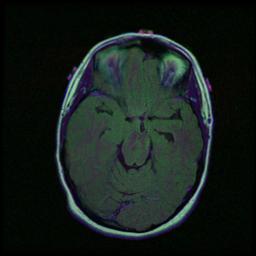

Supplement: S2 Dataset — (ZIP) [file pone.0334447.s002.zip › LGG Segmentation Dataset/train/image/TCGA_DU_6408_19860521_18.jpg]

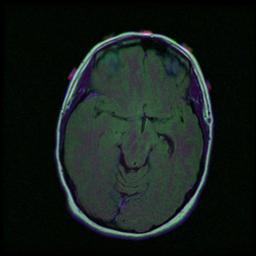

Supplement: S2 Dataset — (ZIP) [file pone.0334447.s002.zip › LGG Segmentation Dataset/train/image/TCGA_DU_6408_19860521_19.jpg]

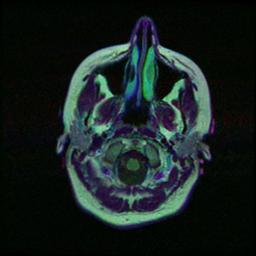

Supplement: S2 Dataset — (ZIP) [file pone.0334447.s002.zip › LGG Segmentation Dataset/train/image/TCGA_DU_6408_19860521_2.jpg]

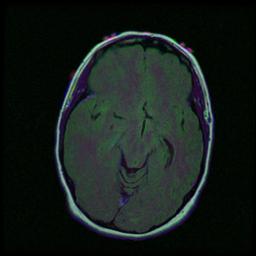

Supplement: S2 Dataset — (ZIP) [file pone.0334447.s002.zip › LGG Segmentation Dataset/train/image/TCGA_DU_6408_19860521_20.jpg]

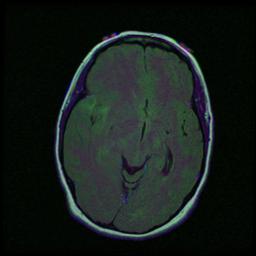

Supplement: S2 Dataset — (ZIP) [file pone.0334447.s002.zip › LGG Segmentation Dataset/train/image/TCGA_DU_6408_19860521_21.jpg]

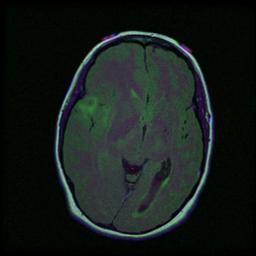

Supplement: S2 Dataset — (ZIP) [file pone.0334447.s002.zip › LGG Segmentation Dataset/train/image/TCGA_DU_6408_19860521_22.jpg]

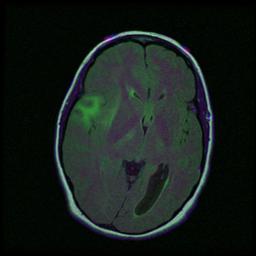

Supplement: S2 Dataset — (ZIP) [file pone.0334447.s002.zip › LGG Segmentation Dataset/train/image/TCGA_DU_6408_19860521_23.jpg]

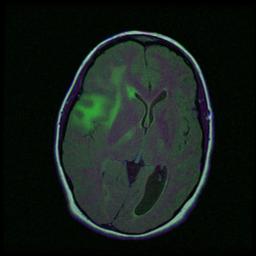

Supplement: S2 Dataset — (ZIP) [file pone.0334447.s002.zip › LGG Segmentation Dataset/train/image/TCGA_DU_6408_19860521_24.jpg]

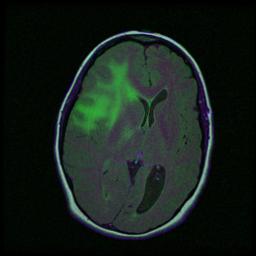

Supplement: S2 Dataset — (ZIP) [file pone.0334447.s002.zip › LGG Segmentation Dataset/train/image/TCGA_DU_6408_19860521_25.jpg]

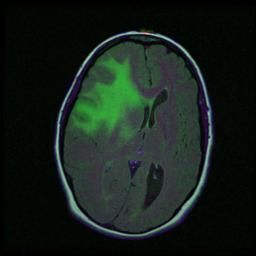

Supplement: S2 Dataset — (ZIP) [file pone.0334447.s002.zip › LGG Segmentation Dataset/train/image/TCGA_DU_6408_19860521_26.jpg]

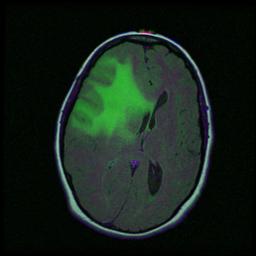

Supplement: S2 Dataset — (ZIP) [file pone.0334447.s002.zip › LGG Segmentation Dataset/train/image/TCGA_DU_6408_19860521_27.jpg]

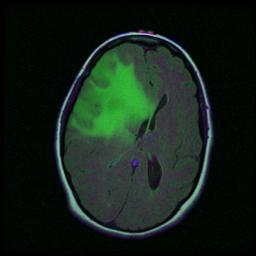

Supplement: S2 Dataset — (ZIP) [file pone.0334447.s002.zip › LGG Segmentation Dataset/train/image/TCGA_DU_6408_19860521_28.jpg]

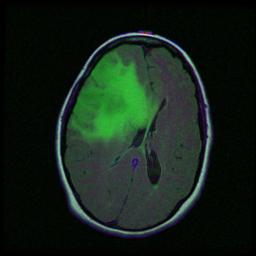

Supplement: S2 Dataset — (ZIP) [file pone.0334447.s002.zip › LGG Segmentation Dataset/train/image/TCGA_DU_6408_19860521_29.jpg]

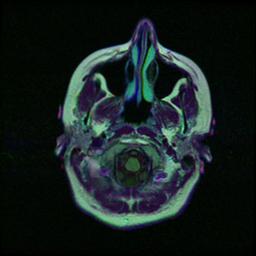

Supplement: S2 Dataset — (ZIP) [file pone.0334447.s002.zip › LGG Segmentation Dataset/train/image/TCGA_DU_6408_19860521_3.jpg]

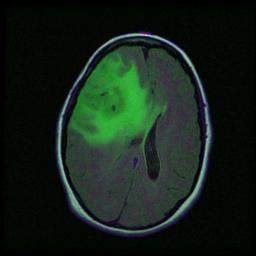

Supplement: S2 Dataset — (ZIP) [file pone.0334447.s002.zip › LGG Segmentation Dataset/train/image/TCGA_DU_6408_19860521_30.jpg]

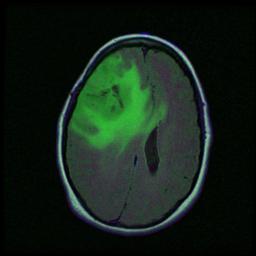

Supplement: S2 Dataset — (ZIP) [file pone.0334447.s002.zip › LGG Segmentation Dataset/train/image/TCGA_DU_6408_19860521_31.jpg]

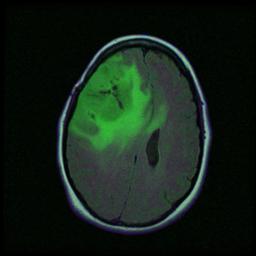

Supplement: S2 Dataset — (ZIP) [file pone.0334447.s002.zip › LGG Segmentation Dataset/train/image/TCGA_DU_6408_19860521_32.jpg]

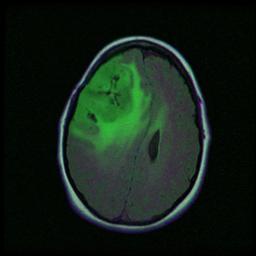

Supplement: S2 Dataset — (ZIP) [file pone.0334447.s002.zip › LGG Segmentation Dataset/train/image/TCGA_DU_6408_19860521_33.jpg]

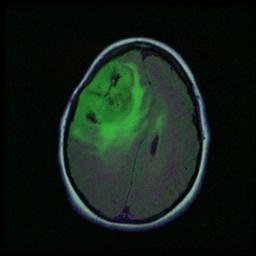

Supplement: S2 Dataset — (ZIP) [file pone.0334447.s002.zip › LGG Segmentation Dataset/train/image/TCGA_DU_6408_19860521_34.jpg]

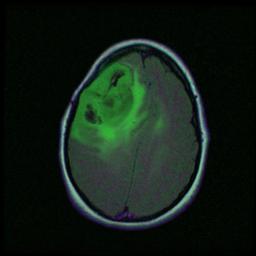

Supplement: S2 Dataset — (ZIP) [file pone.0334447.s002.zip › LGG Segmentation Dataset/train/image/TCGA_DU_6408_19860521_35.jpg]

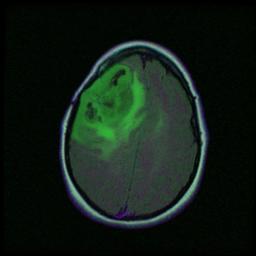

Supplement: S2 Dataset — (ZIP) [file pone.0334447.s002.zip › LGG Segmentation Dataset/train/image/TCGA_DU_6408_19860521_36.jpg]

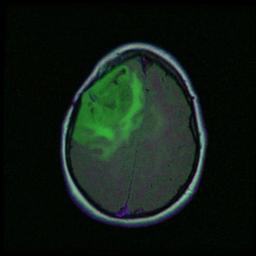

Supplement: S2 Dataset — (ZIP) [file pone.0334447.s002.zip › LGG Segmentation Dataset/train/image/TCGA_DU_6408_19860521_37.jpg]

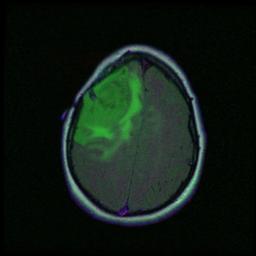

Supplement: S2 Dataset — (ZIP) [file pone.0334447.s002.zip › LGG Segmentation Dataset/train/image/TCGA_DU_6408_19860521_38.jpg]

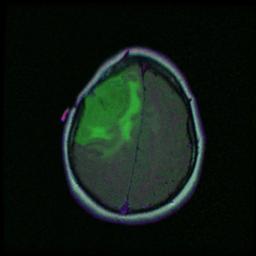

Supplement: S2 Dataset — (ZIP) [file pone.0334447.s002.zip › LGG Segmentation Dataset/train/image/TCGA_DU_6408_19860521_39.jpg]

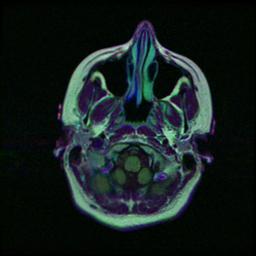

Supplement: S2 Dataset — (ZIP) [file pone.0334447.s002.zip › LGG Segmentation Dataset/train/image/TCGA_DU_6408_19860521_4.jpg]

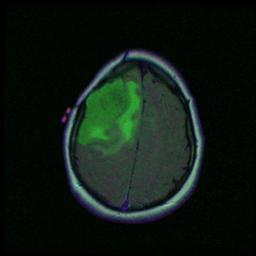

Supplement: S2 Dataset — (ZIP) [file pone.0334447.s002.zip › LGG Segmentation Dataset/train/image/TCGA_DU_6408_19860521_40.jpg]

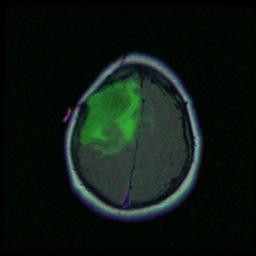

Supplement: S2 Dataset — (ZIP) [file pone.0334447.s002.zip › LGG Segmentation Dataset/train/image/TCGA_DU_6408_19860521_41.jpg]

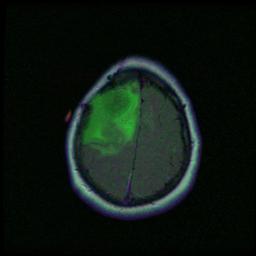

Supplement: S2 Dataset — (ZIP) [file pone.0334447.s002.zip › LGG Segmentation Dataset/train/image/TCGA_DU_6408_19860521_42.jpg]

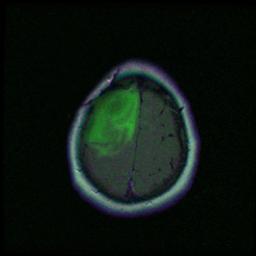

Supplement: S2 Dataset — (ZIP) [file pone.0334447.s002.zip › LGG Segmentation Dataset/train/image/TCGA_DU_6408_19860521_43.jpg]

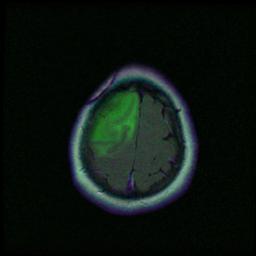

Supplement: S2 Dataset — (ZIP) [file pone.0334447.s002.zip › LGG Segmentation Dataset/train/image/TCGA_DU_6408_19860521_44.jpg]

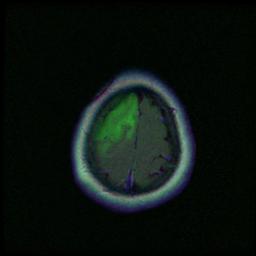

Supplement: S2 Dataset — (ZIP) [file pone.0334447.s002.zip › LGG Segmentation Dataset/train/image/TCGA_DU_6408_19860521_45.jpg]

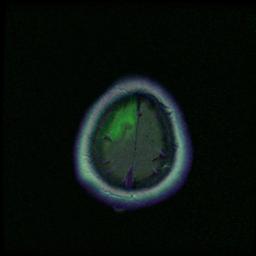

Supplement: S2 Dataset — (ZIP) [file pone.0334447.s002.zip › LGG Segmentation Dataset/train/image/TCGA_DU_6408_19860521_46.jpg]

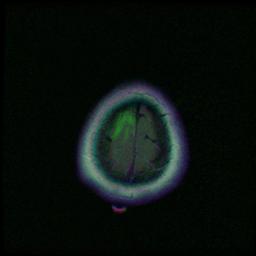

Supplement: S2 Dataset — (ZIP) [file pone.0334447.s002.zip › LGG Segmentation Dataset/train/image/TCGA_DU_6408_19860521_47.jpg]

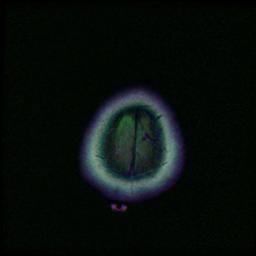

Supplement: S2 Dataset — (ZIP) [file pone.0334447.s002.zip › LGG Segmentation Dataset/train/image/TCGA_DU_6408_19860521_48.jpg]

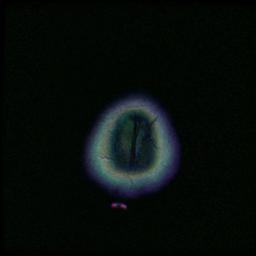

Supplement: S2 Dataset — (ZIP) [file pone.0334447.s002.zip › LGG Segmentation Dataset/train/image/TCGA_DU_6408_19860521_49.jpg]

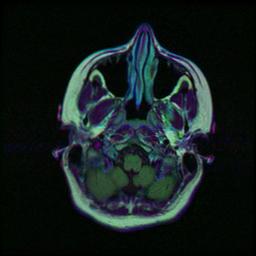

Supplement: S2 Dataset — (ZIP) [file pone.0334447.s002.zip › LGG Segmentation Dataset/train/image/TCGA_DU_6408_19860521_5.jpg]

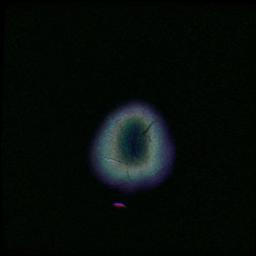

Supplement: S2 Dataset — (ZIP) [file pone.0334447.s002.zip › LGG Segmentation Dataset/train/image/TCGA_DU_6408_19860521_50.jpg]

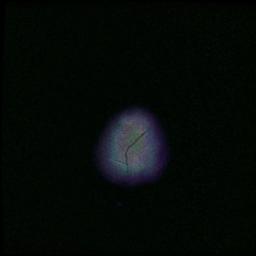

Supplement: S2 Dataset — (ZIP) [file pone.0334447.s002.zip › LGG Segmentation Dataset/train/image/TCGA_DU_6408_19860521_51.jpg]

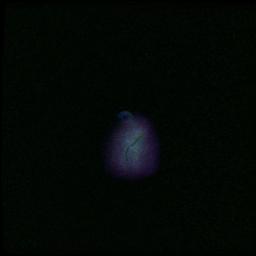

Supplement: S2 Dataset — (ZIP) [file pone.0334447.s002.zip › LGG Segmentation Dataset/train/image/TCGA_DU_6408_19860521_52.jpg]

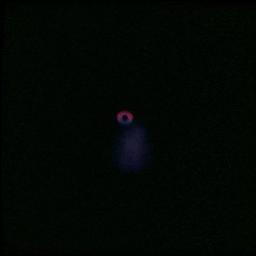

Supplement: S2 Dataset — (ZIP) [file pone.0334447.s002.zip › LGG Segmentation Dataset/train/image/TCGA_DU_6408_19860521_53.jpg]

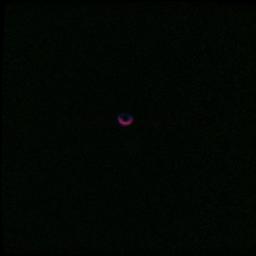

Supplement: S2 Dataset — (ZIP) [file pone.0334447.s002.zip › LGG Segmentation Dataset/train/image/TCGA_DU_6408_19860521_54.jpg]

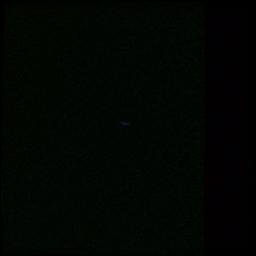

Supplement: S2 Dataset — (ZIP) [file pone.0334447.s002.zip › LGG Segmentation Dataset/train/image/TCGA_DU_6408_19860521_55.jpg]

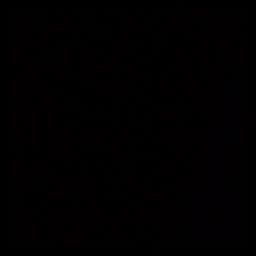

Supplement: S2 Dataset — (ZIP) [file pone.0334447.s002.zip › LGG Segmentation Dataset/train/image/TCGA_DU_6408_19860521_56.jpg]

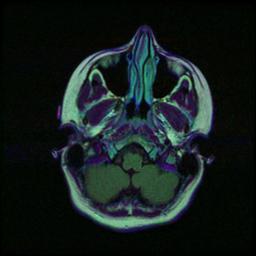

Supplement: S2 Dataset — (ZIP) [file pone.0334447.s002.zip › LGG Segmentation Dataset/train/image/TCGA_DU_6408_19860521_6.jpg]

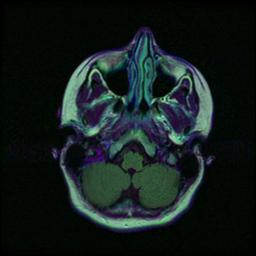

Supplement: S2 Dataset — (ZIP) [file pone.0334447.s002.zip › LGG Segmentation Dataset/train/image/TCGA_DU_6408_19860521_7.jpg]

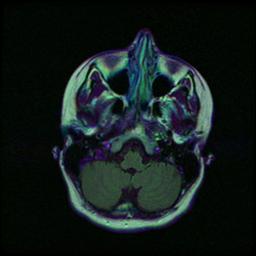

Supplement: S2 Dataset — (ZIP) [file pone.0334447.s002.zip › LGG Segmentation Dataset/train/image/TCGA_DU_6408_19860521_8.jpg]

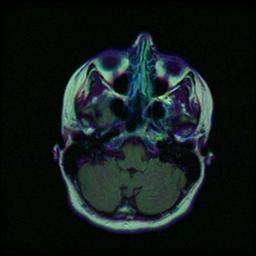

Supplement: S2 Dataset — (ZIP) [file pone.0334447.s002.zip › LGG Segmentation Dataset/train/image/TCGA_DU_6408_19860521_9.jpg]

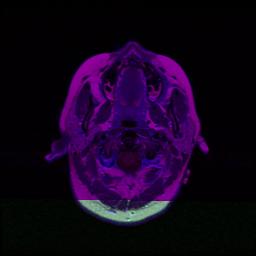

Supplement: S2 Dataset — (ZIP) [file pone.0334447.s002.zip › LGG Segmentation Dataset/train/image/TCGA_DU_7008_19830723_1.jpg]

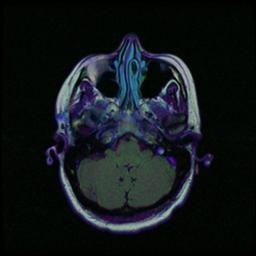

Supplement: S2 Dataset — (ZIP) [file pone.0334447.s002.zip › LGG Segmentation Dataset/train/image/TCGA_DU_7008_19830723_10.jpg]

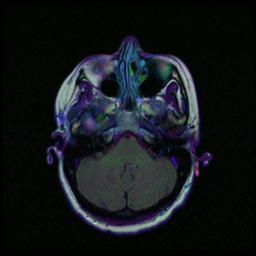

Supplement: S2 Dataset — (ZIP) [file pone.0334447.s002.zip › LGG Segmentation Dataset/train/image/TCGA_DU_7008_19830723_11.jpg]

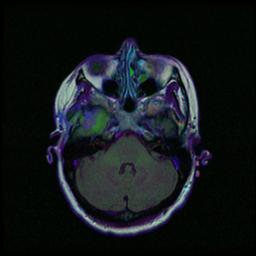

Supplement: S2 Dataset — (ZIP) [file pone.0334447.s002.zip › LGG Segmentation Dataset/train/image/TCGA_DU_7008_19830723_12.jpg]

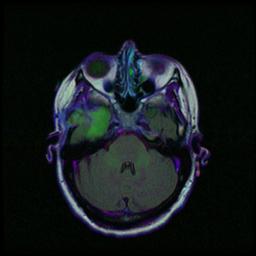

Supplement: S2 Dataset — (ZIP) [file pone.0334447.s002.zip › LGG Segmentation Dataset/train/image/TCGA_DU_7008_19830723_13.jpg]

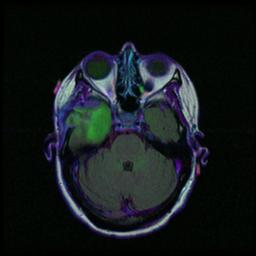

Supplement: S2 Dataset — (ZIP) [file pone.0334447.s002.zip › LGG Segmentation Dataset/train/image/TCGA_DU_7008_19830723_14.jpg]

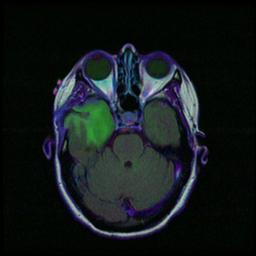

Supplement: S2 Dataset — (ZIP) [file pone.0334447.s002.zip › LGG Segmentation Dataset/train/image/TCGA_DU_7008_19830723_15.jpg]

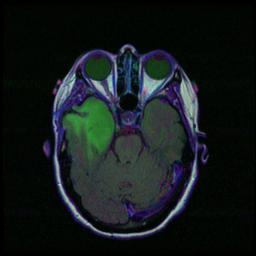

Supplement: S2 Dataset — (ZIP) [file pone.0334447.s002.zip › LGG Segmentation Dataset/train/image/TCGA_DU_7008_19830723_16.jpg]

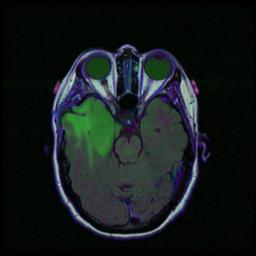

Supplement: S2 Dataset — (ZIP) [file pone.0334447.s002.zip › LGG Segmentation Dataset/train/image/TCGA_DU_7008_19830723_17.jpg]

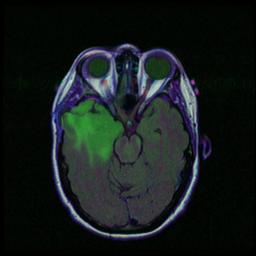

Supplement: S2 Dataset — (ZIP) [file pone.0334447.s002.zip › LGG Segmentation Dataset/train/image/TCGA_DU_7008_19830723_18.jpg]

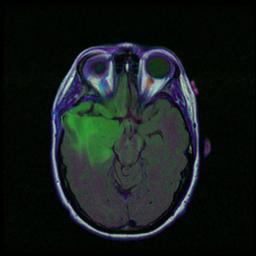

Supplement: S2 Dataset — (ZIP) [file pone.0334447.s002.zip › LGG Segmentation Dataset/train/image/TCGA_DU_7008_19830723_19.jpg]

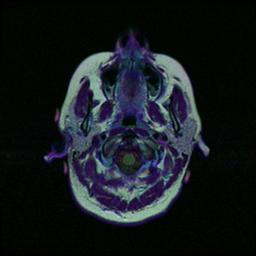

Supplement: S2 Dataset — (ZIP) [file pone.0334447.s002.zip › LGG Segmentation Dataset/train/image/TCGA_DU_7008_19830723_2.jpg]

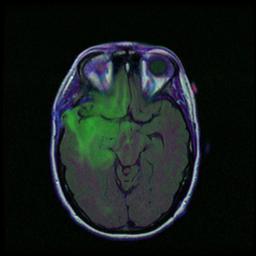

Supplement: S2 Dataset — (ZIP) [file pone.0334447.s002.zip › LGG Segmentation Dataset/train/image/TCGA_DU_7008_19830723_20.jpg]

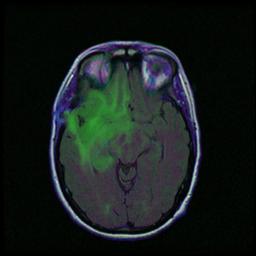

Supplement: S2 Dataset — (ZIP) [file pone.0334447.s002.zip › LGG Segmentation Dataset/train/image/TCGA_DU_7008_19830723_21.jpg]

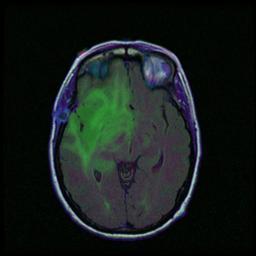

Supplement: S2 Dataset — (ZIP) [file pone.0334447.s002.zip › LGG Segmentation Dataset/train/image/TCGA_DU_7008_19830723_22.jpg]

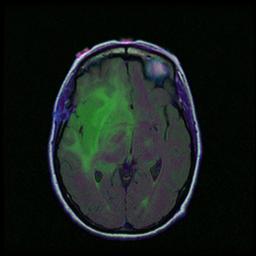

Supplement: S2 Dataset — (ZIP) [file pone.0334447.s002.zip › LGG Segmentation Dataset/train/image/TCGA_DU_7008_19830723_23.jpg]

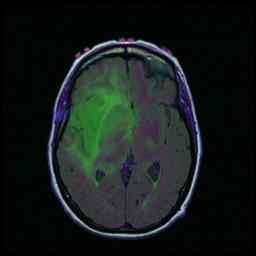

Supplement: S2 Dataset — (ZIP) [file pone.0334447.s002.zip › LGG Segmentation Dataset/train/image/TCGA_DU_7008_19830723_24.jpg]

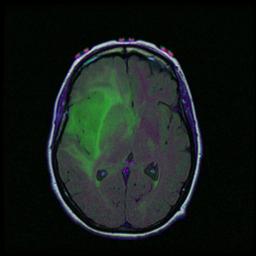

Supplement: S2 Dataset — (ZIP) [file pone.0334447.s002.zip › LGG Segmentation Dataset/train/image/TCGA_DU_7008_19830723_25.jpg]

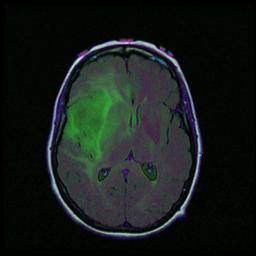

Supplement: S2 Dataset — (ZIP) [file pone.0334447.s002.zip › LGG Segmentation Dataset/train/image/TCGA_DU_7008_19830723_26.jpg]

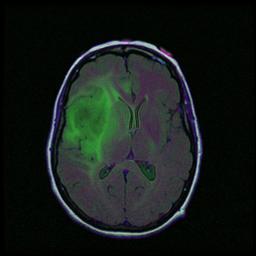

Supplement: S2 Dataset — (ZIP) [file pone.0334447.s002.zip › LGG Segmentation Dataset/train/image/TCGA_DU_7008_19830723_27.jpg]

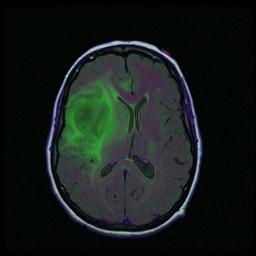

Supplement: S2 Dataset — (ZIP) [file pone.0334447.s002.zip › LGG Segmentation Dataset/train/image/TCGA_DU_7008_19830723_28.jpg]

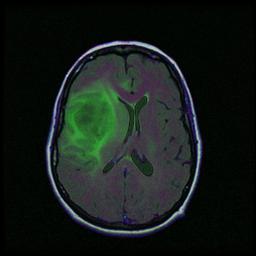

Supplement: S2 Dataset — (ZIP) [file pone.0334447.s002.zip › LGG Segmentation Dataset/train/image/TCGA_DU_7008_19830723_29.jpg]

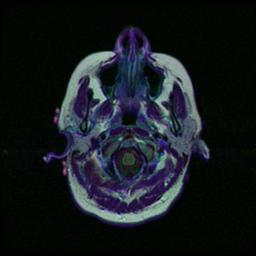

Supplement: S2 Dataset — (ZIP) [file pone.0334447.s002.zip › LGG Segmentation Dataset/train/image/TCGA_DU_7008_19830723_3.jpg]

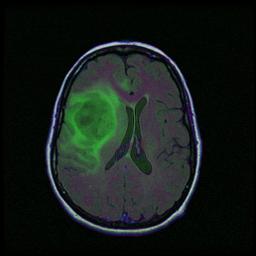

Supplement: S2 Dataset — (ZIP) [file pone.0334447.s002.zip › LGG Segmentation Dataset/train/image/TCGA_DU_7008_19830723_30.jpg]

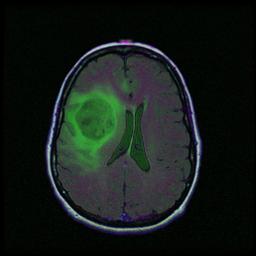

Supplement: S2 Dataset — (ZIP) [file pone.0334447.s002.zip › LGG Segmentation Dataset/train/image/TCGA_DU_7008_19830723_31.jpg]

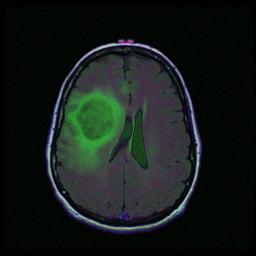

Supplement: S2 Dataset — (ZIP) [file pone.0334447.s002.zip › LGG Segmentation Dataset/train/image/TCGA_DU_7008_19830723_32.jpg]
