# Supplementary figures and images for: Enhanced local feature extraction of lite network with scale-invariant CNN for precise segmentation of small brain tumors in MRI (part 3 of 4)
Source: PLoS One. 2025 Oct 28;20(10):e0334447. doi: 10.1371/journal.pone.0334447 (PMC12561956; doi:10.1371/journal.pone.0334447)

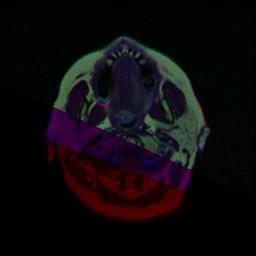

Supplement: S2 Dataset — (ZIP) [file pone.0334447.s002.zip › LGG Segmentation Dataset/train/image/TCGA_DU_6405_19851005_1.jpg]

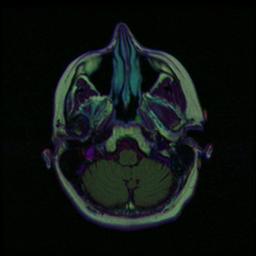

Supplement: S2 Dataset — (ZIP) [file pone.0334447.s002.zip › LGG Segmentation Dataset/train/image/TCGA_DU_6405_19851005_10.jpg]

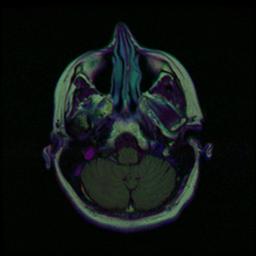

Supplement: S2 Dataset — (ZIP) [file pone.0334447.s002.zip › LGG Segmentation Dataset/train/image/TCGA_DU_6405_19851005_11.jpg]

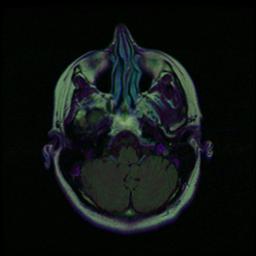

Supplement: S2 Dataset — (ZIP) [file pone.0334447.s002.zip › LGG Segmentation Dataset/train/image/TCGA_DU_6405_19851005_12.jpg]

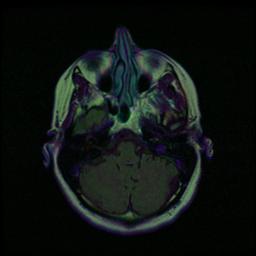

Supplement: S2 Dataset — (ZIP) [file pone.0334447.s002.zip › LGG Segmentation Dataset/train/image/TCGA_DU_6405_19851005_13.jpg]

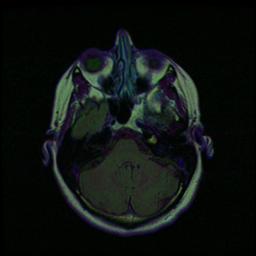

Supplement: S2 Dataset — (ZIP) [file pone.0334447.s002.zip › LGG Segmentation Dataset/train/image/TCGA_DU_6405_19851005_14.jpg]

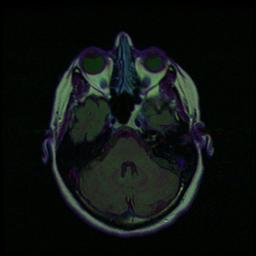

Supplement: S2 Dataset — (ZIP) [file pone.0334447.s002.zip › LGG Segmentation Dataset/train/image/TCGA_DU_6405_19851005_15.jpg]

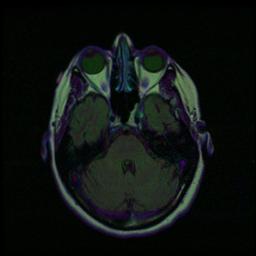

Supplement: S2 Dataset — (ZIP) [file pone.0334447.s002.zip › LGG Segmentation Dataset/train/image/TCGA_DU_6405_19851005_16.jpg]

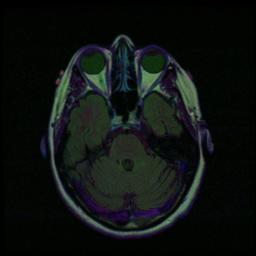

Supplement: S2 Dataset — (ZIP) [file pone.0334447.s002.zip › LGG Segmentation Dataset/train/image/TCGA_DU_6405_19851005_17.jpg]

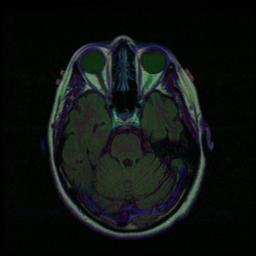

Supplement: S2 Dataset — (ZIP) [file pone.0334447.s002.zip › LGG Segmentation Dataset/train/image/TCGA_DU_6405_19851005_18.jpg]

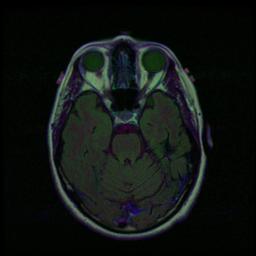

Supplement: S2 Dataset — (ZIP) [file pone.0334447.s002.zip › LGG Segmentation Dataset/train/image/TCGA_DU_6405_19851005_19.jpg]

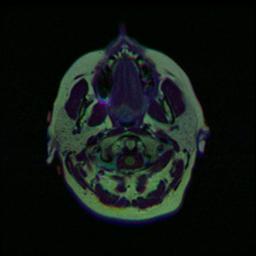

Supplement: S2 Dataset — (ZIP) [file pone.0334447.s002.zip › LGG Segmentation Dataset/train/image/TCGA_DU_6405_19851005_2.jpg]

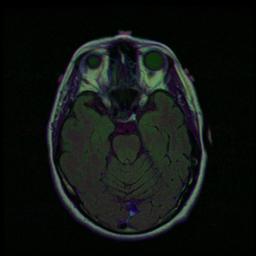

Supplement: S2 Dataset — (ZIP) [file pone.0334447.s002.zip › LGG Segmentation Dataset/train/image/TCGA_DU_6405_19851005_20.jpg]

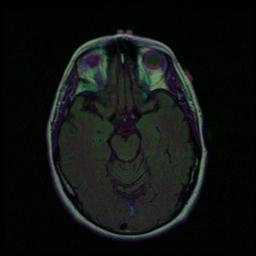

Supplement: S2 Dataset — (ZIP) [file pone.0334447.s002.zip › LGG Segmentation Dataset/train/image/TCGA_DU_6405_19851005_21.jpg]

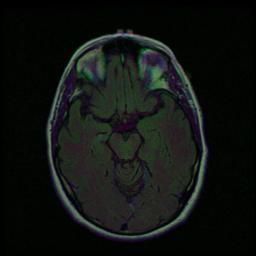

Supplement: S2 Dataset — (ZIP) [file pone.0334447.s002.zip › LGG Segmentation Dataset/train/image/TCGA_DU_6405_19851005_22.jpg]

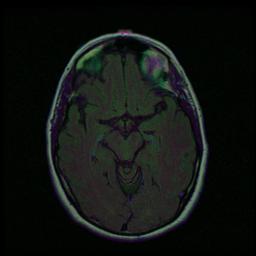

Supplement: S2 Dataset — (ZIP) [file pone.0334447.s002.zip › LGG Segmentation Dataset/train/image/TCGA_DU_6405_19851005_23.jpg]

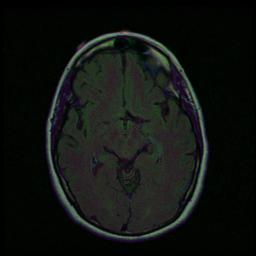

Supplement: S2 Dataset — (ZIP) [file pone.0334447.s002.zip › LGG Segmentation Dataset/train/image/TCGA_DU_6405_19851005_24.jpg]

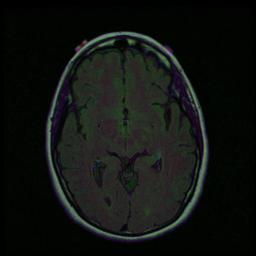

Supplement: S2 Dataset — (ZIP) [file pone.0334447.s002.zip › LGG Segmentation Dataset/train/image/TCGA_DU_6405_19851005_25.jpg]

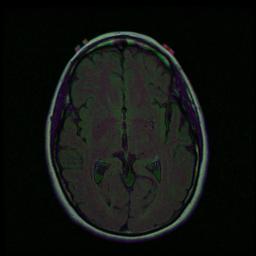

Supplement: S2 Dataset — (ZIP) [file pone.0334447.s002.zip › LGG Segmentation Dataset/train/image/TCGA_DU_6405_19851005_26.jpg]

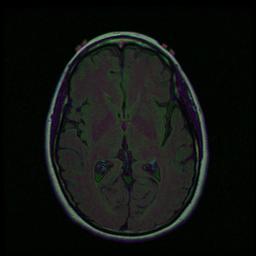

Supplement: S2 Dataset — (ZIP) [file pone.0334447.s002.zip › LGG Segmentation Dataset/train/image/TCGA_DU_6405_19851005_27.jpg]

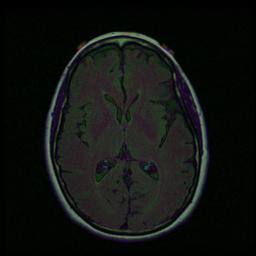

Supplement: S2 Dataset — (ZIP) [file pone.0334447.s002.zip › LGG Segmentation Dataset/train/image/TCGA_DU_6405_19851005_28.jpg]

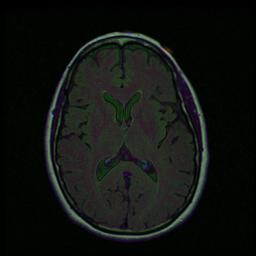

Supplement: S2 Dataset — (ZIP) [file pone.0334447.s002.zip › LGG Segmentation Dataset/train/image/TCGA_DU_6405_19851005_29.jpg]

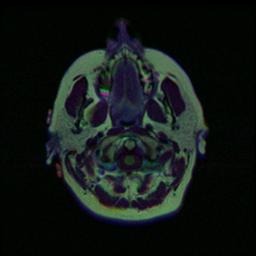

Supplement: S2 Dataset — (ZIP) [file pone.0334447.s002.zip › LGG Segmentation Dataset/train/image/TCGA_DU_6405_19851005_3.jpg]

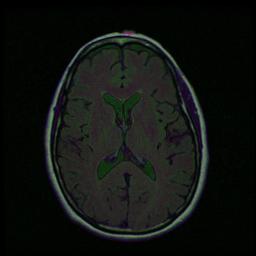

Supplement: S2 Dataset — (ZIP) [file pone.0334447.s002.zip › LGG Segmentation Dataset/train/image/TCGA_DU_6405_19851005_30.jpg]

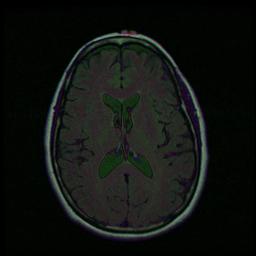

Supplement: S2 Dataset — (ZIP) [file pone.0334447.s002.zip › LGG Segmentation Dataset/train/image/TCGA_DU_6405_19851005_31.jpg]

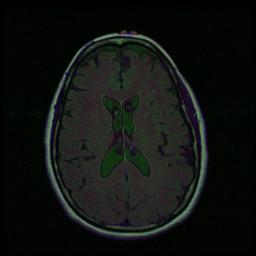

Supplement: S2 Dataset — (ZIP) [file pone.0334447.s002.zip › LGG Segmentation Dataset/train/image/TCGA_DU_6405_19851005_32.jpg]

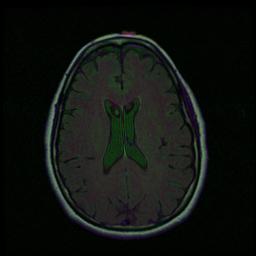

Supplement: S2 Dataset — (ZIP) [file pone.0334447.s002.zip › LGG Segmentation Dataset/train/image/TCGA_DU_6405_19851005_33.jpg]

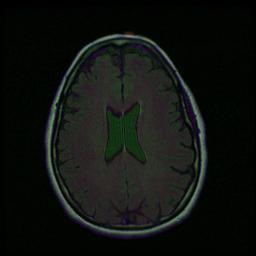

Supplement: S2 Dataset — (ZIP) [file pone.0334447.s002.zip › LGG Segmentation Dataset/train/image/TCGA_DU_6405_19851005_34.jpg]

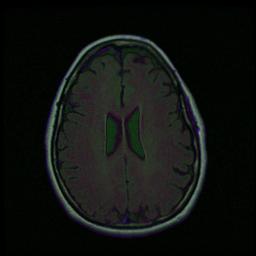

Supplement: S2 Dataset — (ZIP) [file pone.0334447.s002.zip › LGG Segmentation Dataset/train/image/TCGA_DU_6405_19851005_35.jpg]

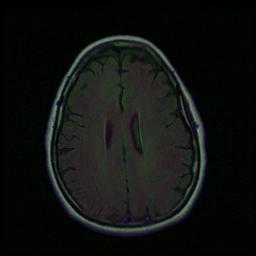

Supplement: S2 Dataset — (ZIP) [file pone.0334447.s002.zip › LGG Segmentation Dataset/train/image/TCGA_DU_6405_19851005_36.jpg]

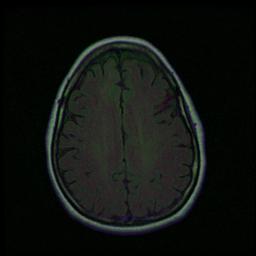

Supplement: S2 Dataset — (ZIP) [file pone.0334447.s002.zip › LGG Segmentation Dataset/train/image/TCGA_DU_6405_19851005_37.jpg]

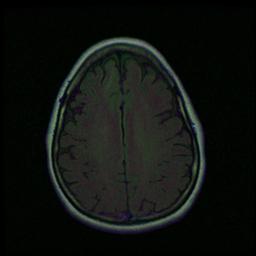

Supplement: S2 Dataset — (ZIP) [file pone.0334447.s002.zip › LGG Segmentation Dataset/train/image/TCGA_DU_6405_19851005_38.jpg]

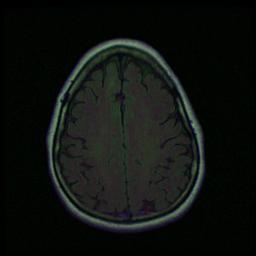

Supplement: S2 Dataset — (ZIP) [file pone.0334447.s002.zip › LGG Segmentation Dataset/train/image/TCGA_DU_6405_19851005_39.jpg]

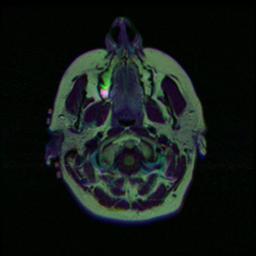

Supplement: S2 Dataset — (ZIP) [file pone.0334447.s002.zip › LGG Segmentation Dataset/train/image/TCGA_DU_6405_19851005_4.jpg]

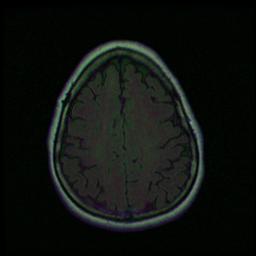

Supplement: S2 Dataset — (ZIP) [file pone.0334447.s002.zip › LGG Segmentation Dataset/train/image/TCGA_DU_6405_19851005_40.jpg]

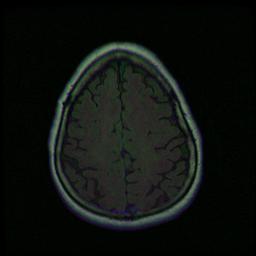

Supplement: S2 Dataset — (ZIP) [file pone.0334447.s002.zip › LGG Segmentation Dataset/train/image/TCGA_DU_6405_19851005_41.jpg]

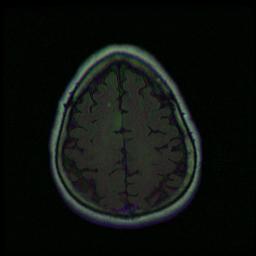

Supplement: S2 Dataset — (ZIP) [file pone.0334447.s002.zip › LGG Segmentation Dataset/train/image/TCGA_DU_6405_19851005_42.jpg]

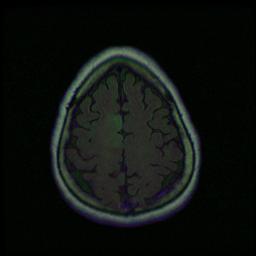

Supplement: S2 Dataset — (ZIP) [file pone.0334447.s002.zip › LGG Segmentation Dataset/train/image/TCGA_DU_6405_19851005_43.jpg]

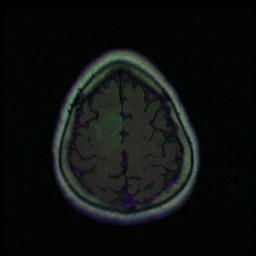

Supplement: S2 Dataset — (ZIP) [file pone.0334447.s002.zip › LGG Segmentation Dataset/train/image/TCGA_DU_6405_19851005_44.jpg]

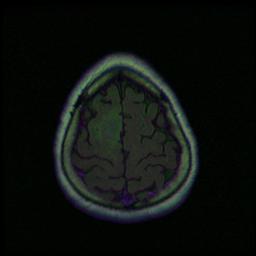

Supplement: S2 Dataset — (ZIP) [file pone.0334447.s002.zip › LGG Segmentation Dataset/train/image/TCGA_DU_6405_19851005_45.jpg]

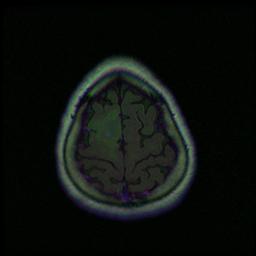

Supplement: S2 Dataset — (ZIP) [file pone.0334447.s002.zip › LGG Segmentation Dataset/train/image/TCGA_DU_6405_19851005_46.jpg]

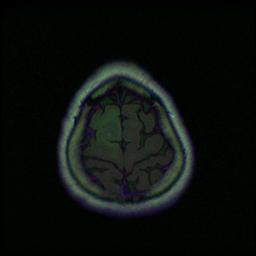

Supplement: S2 Dataset — (ZIP) [file pone.0334447.s002.zip › LGG Segmentation Dataset/train/image/TCGA_DU_6405_19851005_47.jpg]

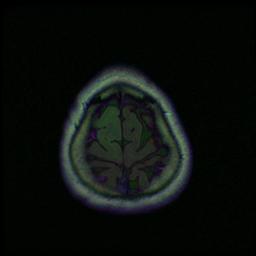

Supplement: S2 Dataset — (ZIP) [file pone.0334447.s002.zip › LGG Segmentation Dataset/train/image/TCGA_DU_6405_19851005_48.jpg]

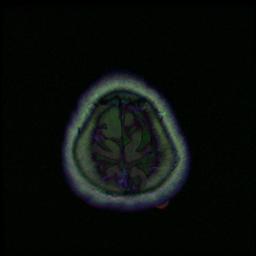

Supplement: S2 Dataset — (ZIP) [file pone.0334447.s002.zip › LGG Segmentation Dataset/train/image/TCGA_DU_6405_19851005_49.jpg]

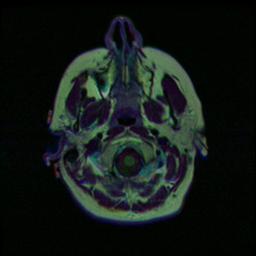

Supplement: S2 Dataset — (ZIP) [file pone.0334447.s002.zip › LGG Segmentation Dataset/train/image/TCGA_DU_6405_19851005_5.jpg]

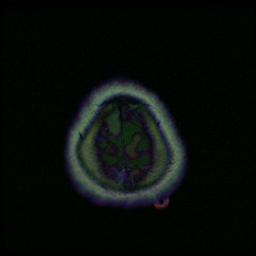

Supplement: S2 Dataset — (ZIP) [file pone.0334447.s002.zip › LGG Segmentation Dataset/train/image/TCGA_DU_6405_19851005_50.jpg]

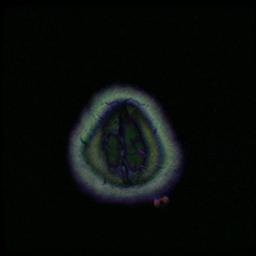

Supplement: S2 Dataset — (ZIP) [file pone.0334447.s002.zip › LGG Segmentation Dataset/train/image/TCGA_DU_6405_19851005_51.jpg]

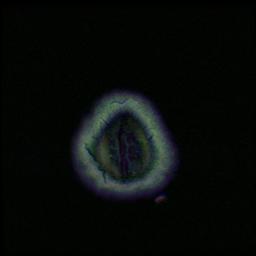

Supplement: S2 Dataset — (ZIP) [file pone.0334447.s002.zip › LGG Segmentation Dataset/train/image/TCGA_DU_6405_19851005_52.jpg]

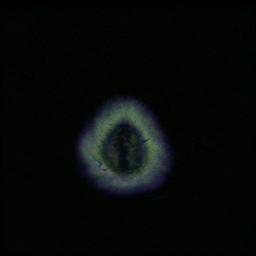

Supplement: S2 Dataset — (ZIP) [file pone.0334447.s002.zip › LGG Segmentation Dataset/train/image/TCGA_DU_6405_19851005_53.jpg]

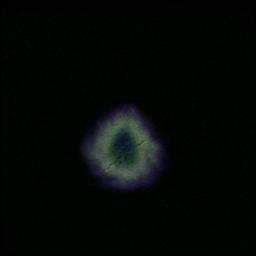

Supplement: S2 Dataset — (ZIP) [file pone.0334447.s002.zip › LGG Segmentation Dataset/train/image/TCGA_DU_6405_19851005_54.jpg]

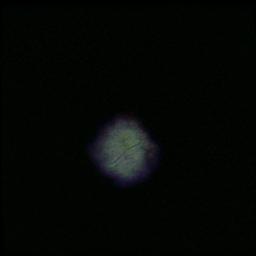

Supplement: S2 Dataset — (ZIP) [file pone.0334447.s002.zip › LGG Segmentation Dataset/train/image/TCGA_DU_6405_19851005_55.jpg]

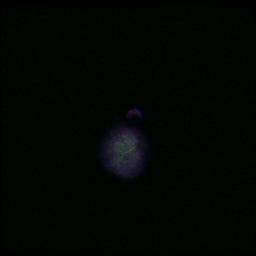

Supplement: S2 Dataset — (ZIP) [file pone.0334447.s002.zip › LGG Segmentation Dataset/train/image/TCGA_DU_6405_19851005_56.jpg]

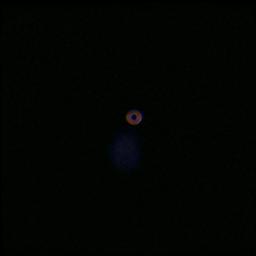

Supplement: S2 Dataset — (ZIP) [file pone.0334447.s002.zip › LGG Segmentation Dataset/train/image/TCGA_DU_6405_19851005_57.jpg]

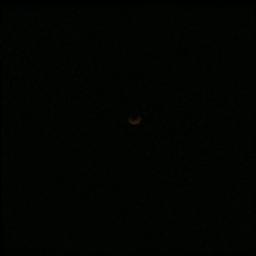

Supplement: S2 Dataset — (ZIP) [file pone.0334447.s002.zip › LGG Segmentation Dataset/train/image/TCGA_DU_6405_19851005_58.jpg]

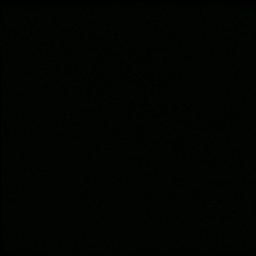

Supplement: S2 Dataset — (ZIP) [file pone.0334447.s002.zip › LGG Segmentation Dataset/train/image/TCGA_DU_6405_19851005_59.jpg]

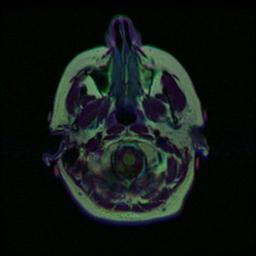

Supplement: S2 Dataset — (ZIP) [file pone.0334447.s002.zip › LGG Segmentation Dataset/train/image/TCGA_DU_6405_19851005_6.jpg]

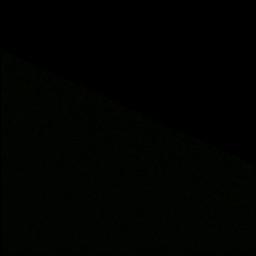

Supplement: S2 Dataset — (ZIP) [file pone.0334447.s002.zip › LGG Segmentation Dataset/train/image/TCGA_DU_6405_19851005_60.jpg]

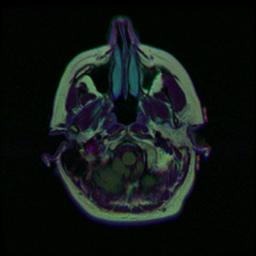

Supplement: S2 Dataset — (ZIP) [file pone.0334447.s002.zip › LGG Segmentation Dataset/train/image/TCGA_DU_6405_19851005_7.jpg]

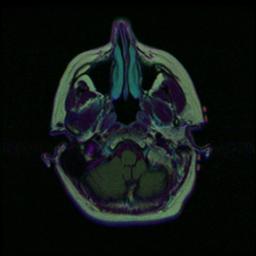

Supplement: S2 Dataset — (ZIP) [file pone.0334447.s002.zip › LGG Segmentation Dataset/train/image/TCGA_DU_6405_19851005_8.jpg]

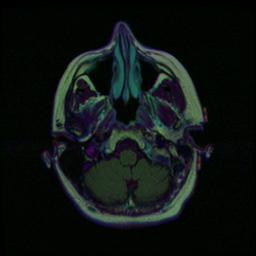

Supplement: S2 Dataset — (ZIP) [file pone.0334447.s002.zip › LGG Segmentation Dataset/train/image/TCGA_DU_6405_19851005_9.jpg]

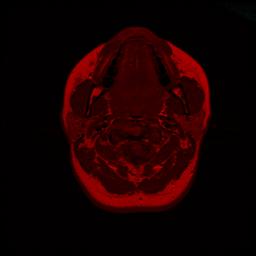

Supplement: S2 Dataset — (ZIP) [file pone.0334447.s002.zip › LGG Segmentation Dataset/train/image/TCGA_DU_6407_19860514_1.jpg]

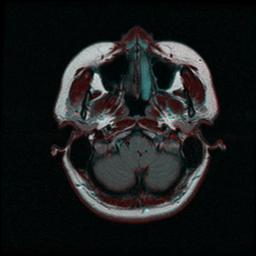

Supplement: S2 Dataset — (ZIP) [file pone.0334447.s002.zip › LGG Segmentation Dataset/train/image/TCGA_DU_6407_19860514_10.jpg]

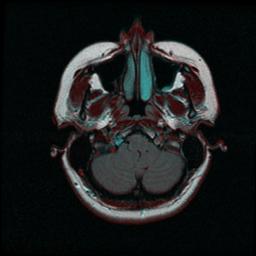

Supplement: S2 Dataset — (ZIP) [file pone.0334447.s002.zip › LGG Segmentation Dataset/train/image/TCGA_DU_6407_19860514_11.jpg]

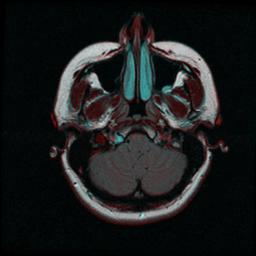

Supplement: S2 Dataset — (ZIP) [file pone.0334447.s002.zip › LGG Segmentation Dataset/train/image/TCGA_DU_6407_19860514_12.jpg]

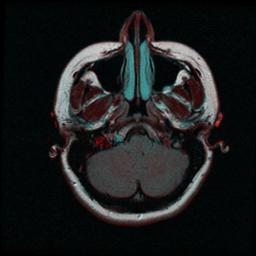

Supplement: S2 Dataset — (ZIP) [file pone.0334447.s002.zip › LGG Segmentation Dataset/train/image/TCGA_DU_6407_19860514_13.jpg]

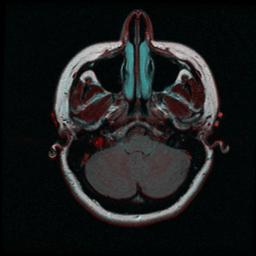

Supplement: S2 Dataset — (ZIP) [file pone.0334447.s002.zip › LGG Segmentation Dataset/train/image/TCGA_DU_6407_19860514_14.jpg]

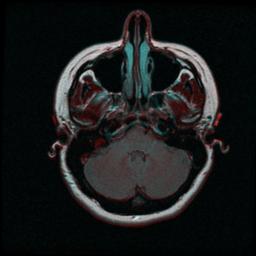

Supplement: S2 Dataset — (ZIP) [file pone.0334447.s002.zip › LGG Segmentation Dataset/train/image/TCGA_DU_6407_19860514_15.jpg]

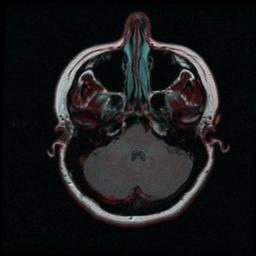

Supplement: S2 Dataset — (ZIP) [file pone.0334447.s002.zip › LGG Segmentation Dataset/train/image/TCGA_DU_6407_19860514_16.jpg]

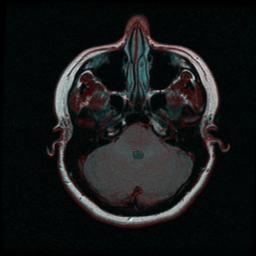

Supplement: S2 Dataset — (ZIP) [file pone.0334447.s002.zip › LGG Segmentation Dataset/train/image/TCGA_DU_6407_19860514_17.jpg]

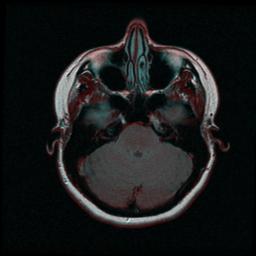

Supplement: S2 Dataset — (ZIP) [file pone.0334447.s002.zip › LGG Segmentation Dataset/train/image/TCGA_DU_6407_19860514_18.jpg]

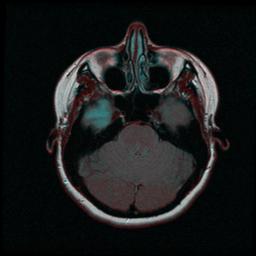

Supplement: S2 Dataset — (ZIP) [file pone.0334447.s002.zip › LGG Segmentation Dataset/train/image/TCGA_DU_6407_19860514_19.jpg]

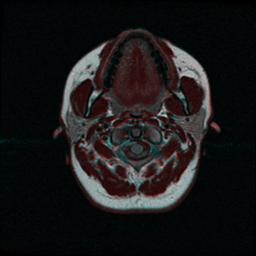

Supplement: S2 Dataset — (ZIP) [file pone.0334447.s002.zip › LGG Segmentation Dataset/train/image/TCGA_DU_6407_19860514_2.jpg]

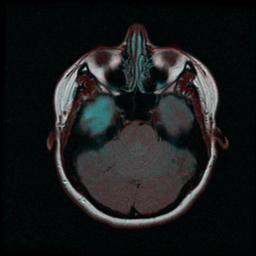

Supplement: S2 Dataset — (ZIP) [file pone.0334447.s002.zip › LGG Segmentation Dataset/train/image/TCGA_DU_6407_19860514_20.jpg]

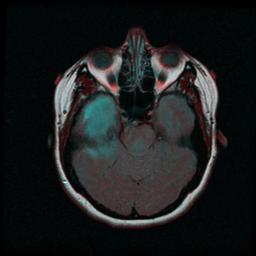

Supplement: S2 Dataset — (ZIP) [file pone.0334447.s002.zip › LGG Segmentation Dataset/train/image/TCGA_DU_6407_19860514_21.jpg]

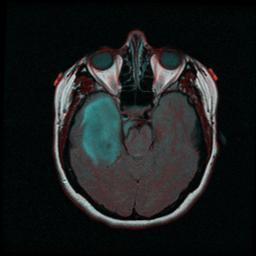

Supplement: S2 Dataset — (ZIP) [file pone.0334447.s002.zip › LGG Segmentation Dataset/train/image/TCGA_DU_6407_19860514_22.jpg]

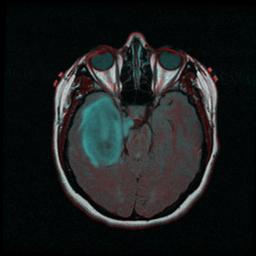

Supplement: S2 Dataset — (ZIP) [file pone.0334447.s002.zip › LGG Segmentation Dataset/train/image/TCGA_DU_6407_19860514_23.jpg]

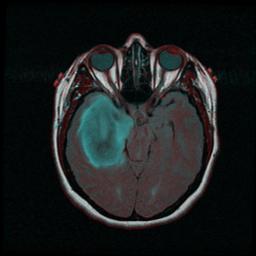

Supplement: S2 Dataset — (ZIP) [file pone.0334447.s002.zip › LGG Segmentation Dataset/train/image/TCGA_DU_6407_19860514_24.jpg]

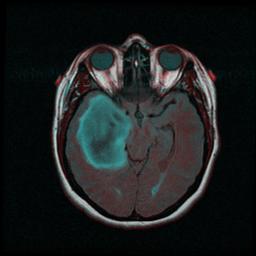

Supplement: S2 Dataset — (ZIP) [file pone.0334447.s002.zip › LGG Segmentation Dataset/train/image/TCGA_DU_6407_19860514_25.jpg]

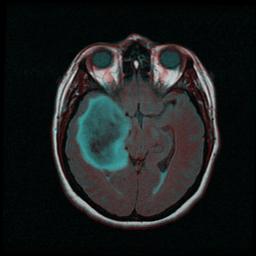

Supplement: S2 Dataset — (ZIP) [file pone.0334447.s002.zip › LGG Segmentation Dataset/train/image/TCGA_DU_6407_19860514_26.jpg]

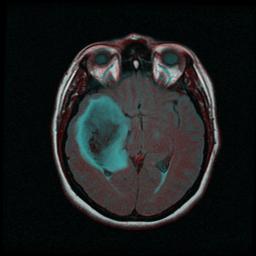

Supplement: S2 Dataset — (ZIP) [file pone.0334447.s002.zip › LGG Segmentation Dataset/train/image/TCGA_DU_6407_19860514_27.jpg]

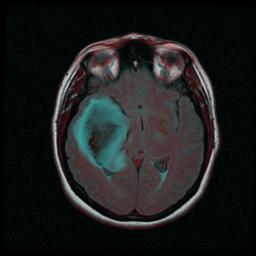

Supplement: S2 Dataset — (ZIP) [file pone.0334447.s002.zip › LGG Segmentation Dataset/train/image/TCGA_DU_6407_19860514_28.jpg]

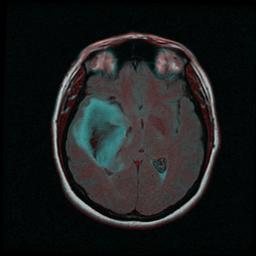

Supplement: S2 Dataset — (ZIP) [file pone.0334447.s002.zip › LGG Segmentation Dataset/train/image/TCGA_DU_6407_19860514_29.jpg]

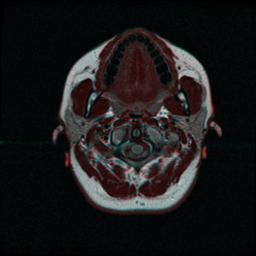

Supplement: S2 Dataset — (ZIP) [file pone.0334447.s002.zip › LGG Segmentation Dataset/train/image/TCGA_DU_6407_19860514_3.jpg]

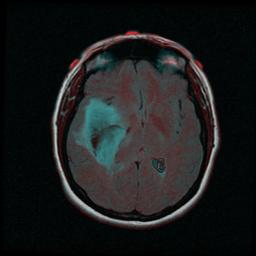

Supplement: S2 Dataset — (ZIP) [file pone.0334447.s002.zip › LGG Segmentation Dataset/train/image/TCGA_DU_6407_19860514_30.jpg]

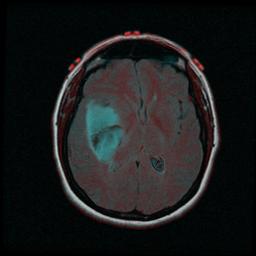

Supplement: S2 Dataset — (ZIP) [file pone.0334447.s002.zip › LGG Segmentation Dataset/train/image/TCGA_DU_6407_19860514_31.jpg]

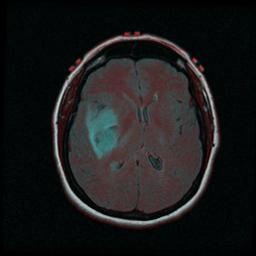

Supplement: S2 Dataset — (ZIP) [file pone.0334447.s002.zip › LGG Segmentation Dataset/train/image/TCGA_DU_6407_19860514_32.jpg]

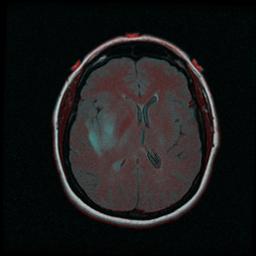

Supplement: S2 Dataset — (ZIP) [file pone.0334447.s002.zip › LGG Segmentation Dataset/train/image/TCGA_DU_6407_19860514_33.jpg]

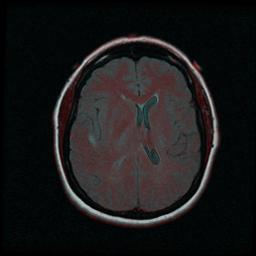

Supplement: S2 Dataset — (ZIP) [file pone.0334447.s002.zip › LGG Segmentation Dataset/train/image/TCGA_DU_6407_19860514_34.jpg]

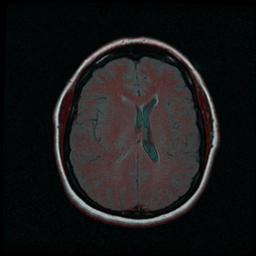

Supplement: S2 Dataset — (ZIP) [file pone.0334447.s002.zip › LGG Segmentation Dataset/train/image/TCGA_DU_6407_19860514_35.jpg]

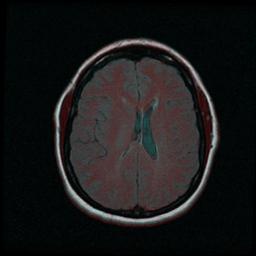

Supplement: S2 Dataset — (ZIP) [file pone.0334447.s002.zip › LGG Segmentation Dataset/train/image/TCGA_DU_6407_19860514_36.jpg]

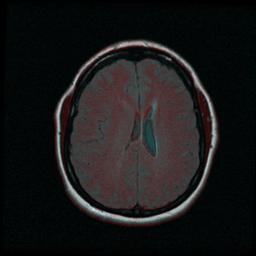

Supplement: S2 Dataset — (ZIP) [file pone.0334447.s002.zip › LGG Segmentation Dataset/train/image/TCGA_DU_6407_19860514_37.jpg]

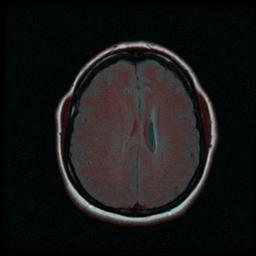

Supplement: S2 Dataset — (ZIP) [file pone.0334447.s002.zip › LGG Segmentation Dataset/train/image/TCGA_DU_6407_19860514_38.jpg]

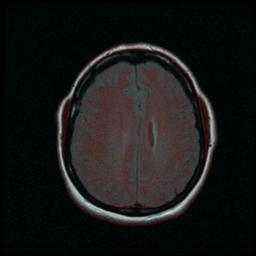

Supplement: S2 Dataset — (ZIP) [file pone.0334447.s002.zip › LGG Segmentation Dataset/train/image/TCGA_DU_6407_19860514_39.jpg]

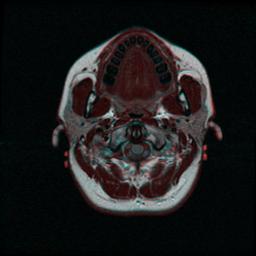

Supplement: S2 Dataset — (ZIP) [file pone.0334447.s002.zip › LGG Segmentation Dataset/train/image/TCGA_DU_6407_19860514_4.jpg]

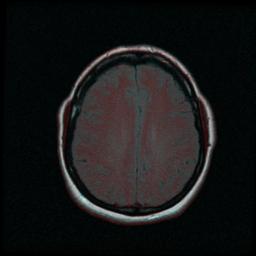

Supplement: S2 Dataset — (ZIP) [file pone.0334447.s002.zip › LGG Segmentation Dataset/train/image/TCGA_DU_6407_19860514_40.jpg]

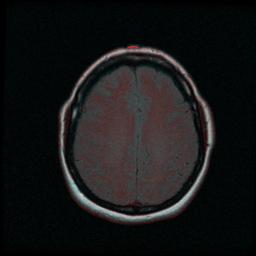

Supplement: S2 Dataset — (ZIP) [file pone.0334447.s002.zip › LGG Segmentation Dataset/train/image/TCGA_DU_6407_19860514_41.jpg]

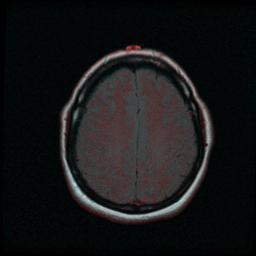

Supplement: S2 Dataset — (ZIP) [file pone.0334447.s002.zip › LGG Segmentation Dataset/train/image/TCGA_DU_6407_19860514_42.jpg]

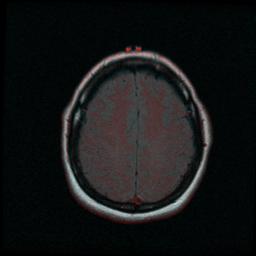

Supplement: S2 Dataset — (ZIP) [file pone.0334447.s002.zip › LGG Segmentation Dataset/train/image/TCGA_DU_6407_19860514_43.jpg]

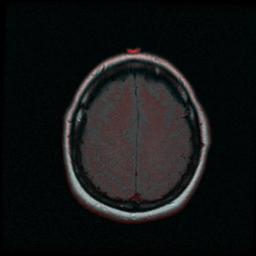

Supplement: S2 Dataset — (ZIP) [file pone.0334447.s002.zip › LGG Segmentation Dataset/train/image/TCGA_DU_6407_19860514_44.jpg]

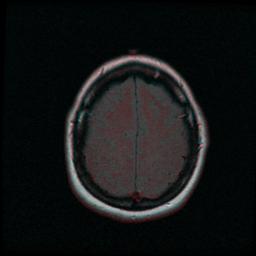

Supplement: S2 Dataset — (ZIP) [file pone.0334447.s002.zip › LGG Segmentation Dataset/train/image/TCGA_DU_6407_19860514_45.jpg]
