# Supplementary material for: Enhanced local feature extraction of lite network with scale-invariant CNN for precise segmentation of small brain tumors in MRI
Source: PLoS One. 2025 Oct 28;20(10):e0334447. doi: 10.1371/journal.pone.0334447 (PMC12561956; doi:10.1371/journal.pone.0334447)
Supplement: Attachment — Submitted filename: response.docx [file pone.0334447.s004.docx]

Reviewer #1: This manuscript presents a lightweight network to perform segmentation task on MRI images utilizing shared CNN layers. The following questions and comments should be addressed during revision:
1. Figure 3, since the authors proposed using shared CNN layers during encoding, why not leverage the parameter saving to explore networks with larger depths? Does increasing the network depth increase the performance of the model?

Response: Our research objective is to enhance the extraction of large-scale detailed features without increasing the network parameters, and even to reduce the network parameters. Although increasing the number of CNN layers would yield better performance, it would lead to more model parameters and higher requirements for the computer’s GPU.

2. Table 1, the overall accuracy metrics are really close for all models reported. The authors should consider repeating the model training process a few times to obtain the mean and standard deviation of the performance metrics to determine if the improvements are statistically significant.

Response:Thank you for your suggestion. We have replaced the GPU with a 4060Ti 16G,

and conducted multiple rounds of model training and continued until the differences between the three metrics of the two best training results were all within 0.5. We then took the average of these two sets of metrics as the final result. The experimental results indeed differed from the previous ones, so we have rewritten the results section.

3. The training time for each model should also be reported.

Response: Thank you for your suggestion. Our model’s advantage lies in its lightweight nature; however, due to the internal implementation of convolutional operations, a lightweight model does not necessarily ensure faster computation.
4. What is the training/testing ratio when the models were trained?

Response: 3,000 images from the LGG Segmentation Dataset were allocated to the training set, while the remaining 929 images were designated for the test set. We have revised the materials subsection and added the following information: For the BraTS21 dataset, the training set contains 580 images and the test set includes 85 images.
5. Figure 4, the meaning of the red bounding box should be explained in the figure caption.

Response: Rectangular bounding boxes have been removed from the new results.
6. A core assumption of this manuscript is the scale invariance of the CNN layers. Have the authors tested this using the datasets available? For example, for the tumor in Figure 4, what happens to all trained model if we crop the image down to a sub-region containing the tumor (resampling back to the same resolution, of course). This effectively changes the scale of the image/tumor. Is the proposed model able to correctly segment out the tumor in this case?

Response: We enable input images of different scales to share the same convolutional neural network (CNN), thereby training the network to recognize features across multiple scales simultaneously. Our assumption is that tumors of varying sizes should all be identifiable by the same network model—analogous to how the human brain can accurately recognize a car both from a distance and up close.

The dataset itself already contains tumors of different sizes, and all models are capable of recognizing them; the only difference lies in the recognition performance among the models. Due to the enhanced depth of large-scale feature extraction in our network, it achieves better performance while having the fewest parameters among all models.

7. Table 3, for the ablation study, another case with just shared CNN layers (without transformers) should be added to see the impact of just the shared CNN layers.

Response: In fact, the change in metric values from the second row ("Transformer + CNN") to the third row ("Transformer + ShareCNN") directly represents the impact solely brought by the shared CNN layers.

Moreover, since convolution primarily captures local features, we specifically apply ShareCNN to large-scale inputs. If we remove the Transformer and use only ShareCNN (which would mean applying ShareCNN to small-scale inputs as well), this would be of little significance.

Reviewer #2: This paper presents LiteMRINet, a lightweight network for segmenting small brain tumors in MRI images. The method introduces a shared 10-layer CNN applied across multiple input scales to enhance local feature extraction while minimizing parameter growth. Additionally, Transformer modules are used on low-resolution feature maps to capture global context. The architecture adopts a U-Net-style decoder and is evaluated on the LGG Segmentation and BraTS21 datasets, demonstrating competitive segmentation performance with significantly fewer parameters compared to existing models. However, before publication, the following concerns should be addressed.


1. Did the study include validation sets? These are typically essential for tuning and early stopping.
Response: In this experiment, we used a training set and a test set. For early stopping, we adopted the criterion that training is halted if the loss function value does not decrease for 20 consecutive epochs.
2. Does the arthor implement the study to test the generalization ability of proposed method?
Response: In our experimental results, the metric values of all models on the test set represent a generalization outcome of the trained models.
3. The usage of “U-Net” and “UNet” should be standardized; “U-Net” is the more widely accepted form.
Response: Thank you for your suggestion. To avoid having two hyphens ("-") in terms like "LeViT-UNet" and "MA-UNet", we have revised all instances of "U-Net" to "UNet".

4. The notation “128×128@3” used to describe data shape is uncommon and should be revised to a more standard format like 128×128×3.
Response: Thank you for your suggestion; we have revised.

5. The first paragraph of the introduction discusses tumor characteristics and clinical context with minimal referencing. I would suggest add more citations to support those statements.

Response: Thank you for your suggestion; we have revised first two paragraph of introduction.
